# Exogenous Biological Renal Support Improves Kidney Function in Mice With Rhabdomyolysis-Induced Acute Kidney Injury

**DOI:** 10.3389/fmed.2021.655787

**Published:** 2021-05-28

**Authors:** Chao Liu, Kun Chi, Xiaodong Geng, Quan Hong, Zhi Mao, Qi Huang, Dong Liu, Yiqin Wang, Ying Zhang, Feihu Zhou, Guangyan Cai, Xiangmei Chen, Xuefeng Sun

**Affiliations:** ^1^Medical School of Chinese People's Liberation Army (PLA), Beijing, China; ^2^State Key Laboratory of Kidney Diseases, Department of Nephrology, National Clinical Research Center for Kidney Diseases, Chinese People's Liberation Army (PLA) Institute of Nephrology, Chinese People's Liberation Army (PLA) General Hospital, Beijing, China; ^3^Department of Critical Care Medicine, Chinese People's Liberation Army (PLA) General Hospital, Beijing, China; ^4^Department of Nephrology, Beijing Tiantan Hospital, Capital Medical University, Beijing, China; ^5^Department of Nephrology, Air Force Medical Center, People's Liberation Army (PLA), Beijing, China; ^6^Department of Ultrasound, Chinese People's Liberation Army (PLA) General Hospital, Beijing, China

**Keywords:** parabiosis model, exogenous biological renal support, rhabdomyolysis, acute kidney injury, bioinformatics analysis

## Abstract

**Background:** Rhabdomyolysis (RM) is a clinical syndrome characterized by breakdown of skeletal muscle fibers and release of their contents into the circulation. Myoglobin-induced acute kidney injury (AKI) is one of the most severe complications of RM. Based on our previous research, exogenous biological renal support alleviates renal ischemia–reperfusion injury in elderly mice. This study aimed to determine whether exogenous biological renal support promotes renal recovery from RM-induced AKI and to preliminarily explore the mechanisms involved.

**Methods:** A parabiosis animal model was established to investigate the effects of exogenous biological renal support on RM-induced AKI. Mice were divided into three groups: the control group (in which mice were injected with sterile saline), the RM group (in which mice were injected with 8 mL/kg glycerol), and the parabiosis + RM group (in which recipient mice were injected with glycerol 3 weeks after parabiosis model establishment). Blood samples and kidney tissue were collected for further processing 48 h after RM induction. Bioinformatics analysis was conducted via Gene Ontology analysis, Kyoto Encyclopedia of Genes and Genomes pathway analysis, functional enrichment analysis, and clustering analysis.

**Results:** No mice died within 48 h after the procedure. Exogenous biological renal support attenuated the histological and functional deterioration in mice with RM-induced AKI. Bioinformatics analysis identified key pathways and proteins involved in this process. We further demonstrated that exogenous biological renal support ameliorated AKI through multiple mechanisms, including by suppressing the complement system; attenuating oxidative stress, inflammation, and cell death; and increasing proliferation.

**Conclusions:** Exogenous biological renal support provided by parabiosis can improve renal function in RM-induced AKI by suppressing the complement system; decreasing oxidative stress, inflammation, and cell death; and promoting tubular cell proliferation. Our study provides basic research evidence for the use of bioartificial kidneys to treat RM-induced AKI.

## Background

Rhabdomyolysis (RM) is a clinical syndrome characterized by breakdown of skeletal muscle fibers and release of their contents into the circulation (1, 2). It can be caused by many factors, such as trauma (3, 4), heat exposure (5), endurance running (6), drugs (7), and viruses [such as SARS-CoV-2 (severe acute respiratory syndrome coronavirus 2)] (8–10). Myoglobin (MYO)–induced acute kidney injury (AKI) is one of the most severe complications of RM (11). MYO and its oxygen-carrying moiety heme play a key role in the development of AKI. AKI can cause renal tubular obstruction and renal vasoconstriction and can directly activate oxidative stress, lipid peroxidation, and macrophages, thus injuring proximal tubular cells (12–14).

Renal replacement therapy (RRT), in which a high-permeability membrane is used to eliminate circulating MYO, is often used in the treatment of RM-induced AKI. However, RRT is a non-specific treatment method that removes blood solutes; it cannot regulate inflammation or promote repair of damaged renal tubules. Moreover, previous research has shown that RRT does not significantly reduce mortality or improve renal repair in AKI patients, while artificial biology methods can reduce mortality in patients with AKI (15–17). Mouse parabiosis models have been established that involve a shared circulatory system; the linked mice share circulating antigens and do not exhibit adverse immune reactions (18, 19). Such models have been used in several physiological studies on various topics, such as kidney hypertension (20), hematopoietic stem cell migration (21), neurodegenerative disease (22), and lymphocyte trafficking (23). Our previous research used a parabiosis model to show that exogenous biological renal support may attenuate inflammation and apoptosis and increase proliferation in mice with ischemia–reperfusion injury (IRI) (17, 24).

In this study, we established a parabiosis model in mice and then used glycerol injections to induce AKI. To study the therapeutic effects of exogenous biological renal support on RM-induced AKI, proteomic analysis was used to screen and study changes in protein expression and key pathways and to verify the effects on the complement system, oxidative stress, inflammation, cell death, and renal tubular epithelial cell proliferation. We aimed to determine whether exogenous biological renal support promotes renal recovery from RM-induced AKI and to preliminarily explore the mechanisms involved.

## Materials and Methods

### Experimental Animals

All animal protocols were approved by the Animal Ethics Committee of the Chinese PLA General Hospital. Male C57BL/6 mice (8–12 weeks old) were purchased from the Si Bei Fu Laboratory Animal Company (Beijing, China). Male Actb-green fluorescent protein (GFP)/C57/BL6 transgenic mice expressing GFP were purchased from the Model Animal Research Center of Chinese Nanjing University (Nanjing, China). All animals were given free access to food and water.

Parabiosis was performed based on the methods developed by Donskoy and Goldschneider (23). The sharing of circulation between the two mice was verified as described in our previous research (24, 25). Briefly, after anesthetization (intraperitoneal injection of 1% pentobarbital sodium at a dose of 30 mg/kg) and sterilization, the skin and subcutaneous tissue of two mice were cut to expose the subcutaneous muscle. The chest and back muscles of the donor mouse were separated and sutured to the chest and back muscles of the recipient mouse. Then, the edges of the skin were sutured. The details of this procedure are described Supplementary Figure 1.

After 3 weeks of parabiosis, the RM model was induced as previously reported (26). The mice were deprived of water for 24 h and then administered diluted glycerol (50% vol/vol in sterile saline) in each hindlimb muscle at a dose of 8 mL/kg following mild sedation with pentobarbital. Blood samples and kidney tissue were collected for further processing 48 h after induction of RM.

The mice were divided into three groups: the sham group (9 mice), in which the mice were injected with sterile saline; the RM group (9 mice), in which the mice were injected with glycerol; and the parabiosis + RM group (18 mice, 9 pairs). Three weeks after the parabiosis model was established, the recipient mouse was administered glycerol; this mouse was defined as the P_RM_R. The other mouse in the parabiosis model supplied exogenous biological renal support and was defined as the P_RM_S.

### Verification of the Establishment of Shared Circulation in Parabiotic Mice

GFP-expressing mice and wild-type mice were used for parabiosis. The successful establishment of shared blood circulation was verified by three methods from our previous studies (24): (1) GFP detection with a peripheral blood smear test, (2) GFP-positive cell ratio measurement via flow cytometry, and (3) small animal *in vivo* imaging. For further details on the methods (Supplementary Figure 2).

### Serum Biochemistry Analysis

An automatic biochemistry analyzer (Cobas 8000; Roche, Mannheim, Germany) was used to measure blood urea nitrogen (BUN) and serum creatinine.

### Renal Morphology

All tissue sections were independently evaluated by two investigators (X.D.G. and Q.H.) in a blinded manner. Kidneys were fixed in 4% paraformaldehyde solution overnight, embedded in paraffin, sectioned at a thickness of 3 μm, and stained with periodic acid–Schiff. Renal tubular injury was scored by counting the percentage of tubules that displayed cellular necrosis, loss of the brush border, cast formation, and tubule dilatation. Zero represents normal histology, and 1–5 represent ≤ 10, ≤ 25, ≤ 50, ≤ 75, and >75% abnormal histology, respectively (24).

### Measurements of Superoxide Dismutase, Glutathione Peroxidase, Malondialdehyde, Creatine Kinase, and Protein Carbonyl Levels in Kidney Samples

A 10% homogenate was prepared from kidney tissue and centrifuged at 730 × *g* at 4°C for 15 min to obtain the supernatant. Total superoxide dismutase (SOD), glutathione peroxidase (GSH), malondialdehyde (MDA), creatine kinase (CK), and protein carbonyl (PC) levels were measured with commercial kits according to the manufacturer's instructions (Beijing Jinenlai Biochemistry Co., Beijing, China).

### Measurements of MYO, CK, Neutrophil Gelatinase-Associated Lipid Carrier Protein, Tumor Necrosis Factor α, and Serum Amyloid A1 and A2 Levels in Serum Samples

Mouse plasma was centrifuged at 730 × g at 4°C for 10 min. Then, the upper serum layer was collected. The levels of MYO, CK, neutrophil gelatinase-associated lipid carrier protein (NGAL), tumor necrosis factor α (TNF-α), and serum amyloid A1 and A2 (SAA1 and SAA2) were measured with commercial kits according to the manufacturer's instructions (Beijing Jinenlai Biochemistry Co., Beijing, China).

### Western Blot Analysis

Briefly, 50–100 μg of protein from each sample was separated by sodium dodecyl sulfate–polyacrylamide gel electrophoresis. The membrane was incubated with the following antibodies at 4°C overnight: anti–cleaved caspase-3 (9664, 1:500; Cell Signaling Technology), anti-Bcl2 (2876, 1:1,000; Cell Signaling Technology), anti–cyclin D1 (60186, 1:1,000; Proteintech), anti–cyclin E1 (11554, 1:1,000; Proteintech), and anti–β-actin (0061R, 1:2,000; Beijing Biosynthesis Biotechnology Co.). The blots were probed with horseradish peroxidase–conjugated goat anti-rabbit or goat anti-mouse immunoglobulin G (IgG) (1:1,000; Santa Cruz Biotechnology). Immunoreactive bands were visualized by enhanced chemiluminescence, and the blot signals were analyzed with the image analysis software ImageJ 1.52.

### TUNEL Staining

Kidney tissue was fixed in 10% formalin overnight, dehydrated, embedded in paraffin, and cut into 3-μm-thick sections. The sections were placed on numbered polylysine-coated glass slides. Terminal deoxynucleotidyl transferase-mediated deoxyuridine triphosphate nick end labeling (TUNEL)–positive cells, which were stained brown, were counted under 200 × magnification. Nuclei were stained with hematoxylin to observe the characteristics of TUNEL-positive cells. Six to eight fields per section and two to three sections per kidney were examined in each experiment. We calculated the percentage of TUNEL-positive cells relative to the total number of renal tubular cells as the apoptosis rate. The TUNEL assay was performed according to the manufacturer's instructions (Merck Millipore, Billerica, MA, USA).

### Immunohistochemistry

Immunohistochemical staining for the detection of CD3 and proliferating cell nuclear antigen (PCNA) in renal tissue was performed on formaldehyde-fixed and paraffin-embedded tissues using the avidin–biotin–immunoperoxidase method. As described in our previous research (17, 24), after antigen retrieval, the sections were incubated with anti-CD3 (5690, 1:100; Abcam) or anti-PCNA antibodies (18197, 1:1,000; Abcam) overnight at 4°C, immersed in biotin-conjugated goat anti-rabbit IgG for 40 min, and finally incubated with streptavidin-conjugated peroxidase for 30 min. Five fields on each of three slides per animal were observed under a microscope.

### Immunofluorescence

Immunofluorescence staining for the detection of complement 3 (C3) in renal tissue was performed on formaldehyde-fixed and paraffin-embedded tissues. The slides were air-dried, fixed with methanol/acetone for 10 min, and treated with a fluorescein isothiocyanate–conjugated anti-C3 antibody (21337, 1:500; Proteintech) at room temperature for 1 h. Nuclei were counterstained with DAPI. Five fields on each of three slides per animal were randomly selected for visualization, and analysis was performed using ImageJ software.

### Proteomic Sample Extraction and Liquid Chromatography–Tandem Mass Spectrometry Analysis

Peripheral blood and kidney tissue were collected and digested with trypsin. After isobaric tags for relative and absolute quantitation (iTRAQ) labeling, the tryptic peptides were fractionated with high-pH reverse-phase high-performance liquid chromatography using a Thermo BetaSil C18 column (5-μm particles, 10-mm inner diameter, and 250-mm length), and then the peptides were subjected to nanospray ionization followed by tandem mass spectrometry (MS/MS) in a Q Exactive™ Plus (Thermo) coupled online to an ultraperformance liquid chromatography platform. The resulting MS/MS data were processed using the MaxQuant search engine (v.1.5.2.8). The details of these procedures are provided in Supplementary File 1.

### Bioinformatics Analysis

Bioinformatics analysis was conducted via Gene Ontology (GO) analysis, Kyoto Encyclopedia of Genes and Genomes (KEGG) pathway analysis, functional enrichment analysis, and clustering analysis. The details of the software and analysis parameters are provided in Supplementary File 1. A positive or negative fold change of 1.3 and a *P*-value lower than 0.05 were chosen as the thresholds for identification of significantly upregulated or downregulated proteins, respectively.

### Statistical Analyses

All data were analyzed using R 3.6.1 software. The data are expressed as the mean and standard deviation (SD). Statistical significance was determined using two-way analysis of variance or Student *t*-test. Categorical variables are expressed as proportions and were compared using the χ^2^ test. Statistical graphs were produced with GraphPad Prism (GraphPad Software Inc., La Jolla, CA). *P* < 0.05 was considered to indicate statistical significance.

## Results

### Verification of Cross-Circulation in the Parabiosis Model

Male wild-type C57BL/6 mice and C57BL/6-TgN (ACTb-EGFP) transgenic mice were used for parabiosis, and the establishment of shared circulation was confirmed among parabiotic pairs 3 weeks after the parabiosis surgery. The three sets of results consistently demonstrated that 3 weeks after parabiosis surgery, the donor and recipient mice shared blood circulation. The results are shown in Supplementary Figure 2 and Supplementary File 2.

### Exogenous Biological Renal Support Attenuated Histological and Functional Deterioration in Mice With RM-Induced AKI

**Figure 1:**
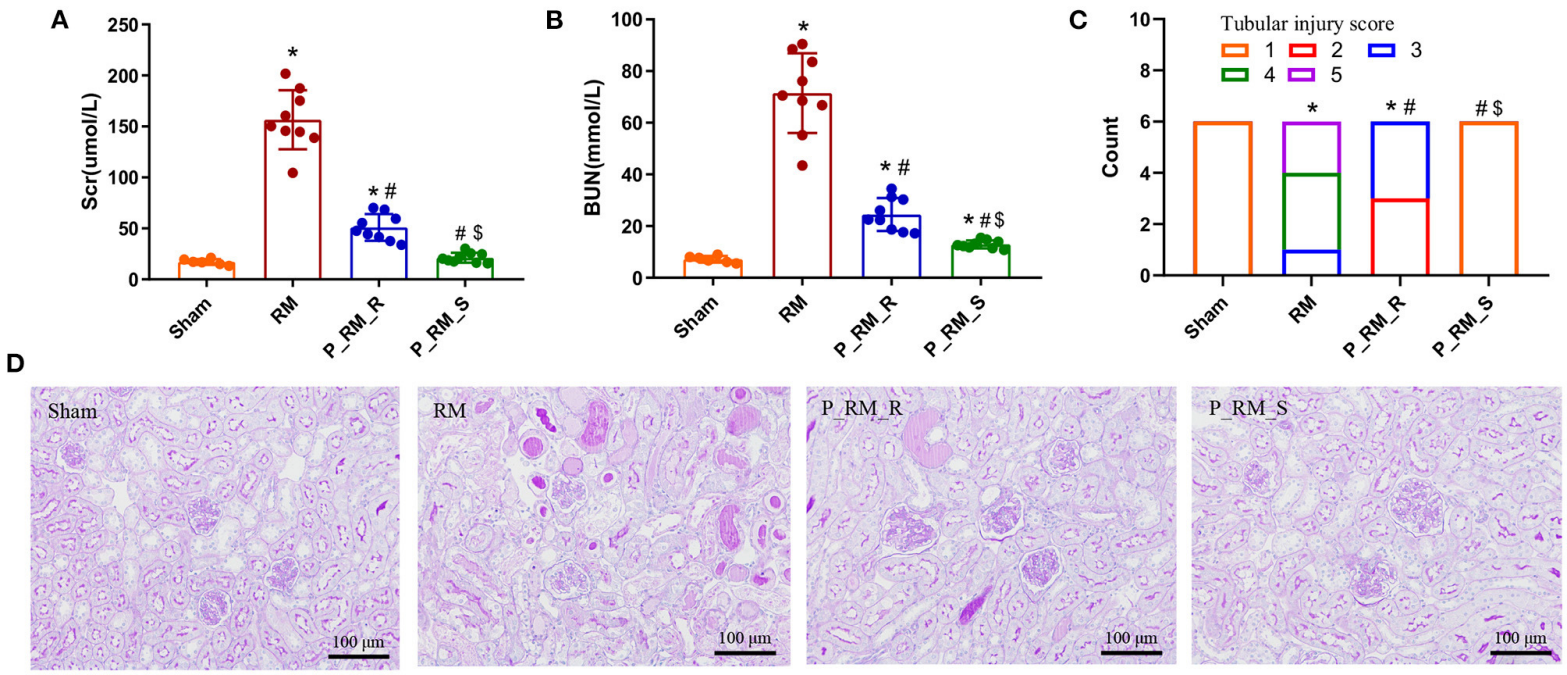
Exogenous biological renal support attenuated the histological and functional deterioration in RM-induced AKI. **(A)** Scr levels in the four groups. **(B)** BUN levels in the four groups. **(C)** Renal tubular injury scores. **(D)** Representative photographs of kidney sections stained with periodic acid–Schiff (200 × magnification). The values are presented as means ± SD. Scr, serum creatinine; BUN, blood urea nitrogen; RM, rhabdomyolysis; P_RM_R, the mouse in the parabiosis model administered glycerol; P_RM_S, the other mouse in the parabiosis model that supplied the exogenous biological renal support. *P < 0.05 vs. the sham group; ^#^*P* < 0.05 vs. the RM group; ^$^*P* < 0.05 vs. the P_RM_R group.

None of the mice died within 48 h following the procedure. Glycerol administration resulted in significant increases in Scr and BUN levels in the RM group compared with the parabiosis + RM and sham groups. Sham mice did not show any significant tubular damage. RM mice showed tubular brush border loss, cast formation, tubular dilatation, and tubular necrosis, accompanied by increased renal tubular injury scores. Parabiosis + RM mice had significantly improved renal histological injury (Figure 1).

### Proteomic Analysis Results

We first analyzed the proteome quality of three replicates. Principal component analysis and Pearson correlation coefficient analysis suggested that the proteome quality was suitable for subsequent analysis (Supplementary Figure 3).

A total of 5,887 identified proteins were found in the kidney tissue sample proteome, among which 5,136 proteins were quantified. In the serum sample proteome, 1,102 identified proteins were found, among which 962 proteins were quantified. The upregulated and downregulated proteins in the different groups are shown in Supplementary Figure 4.

**Figure 2:**
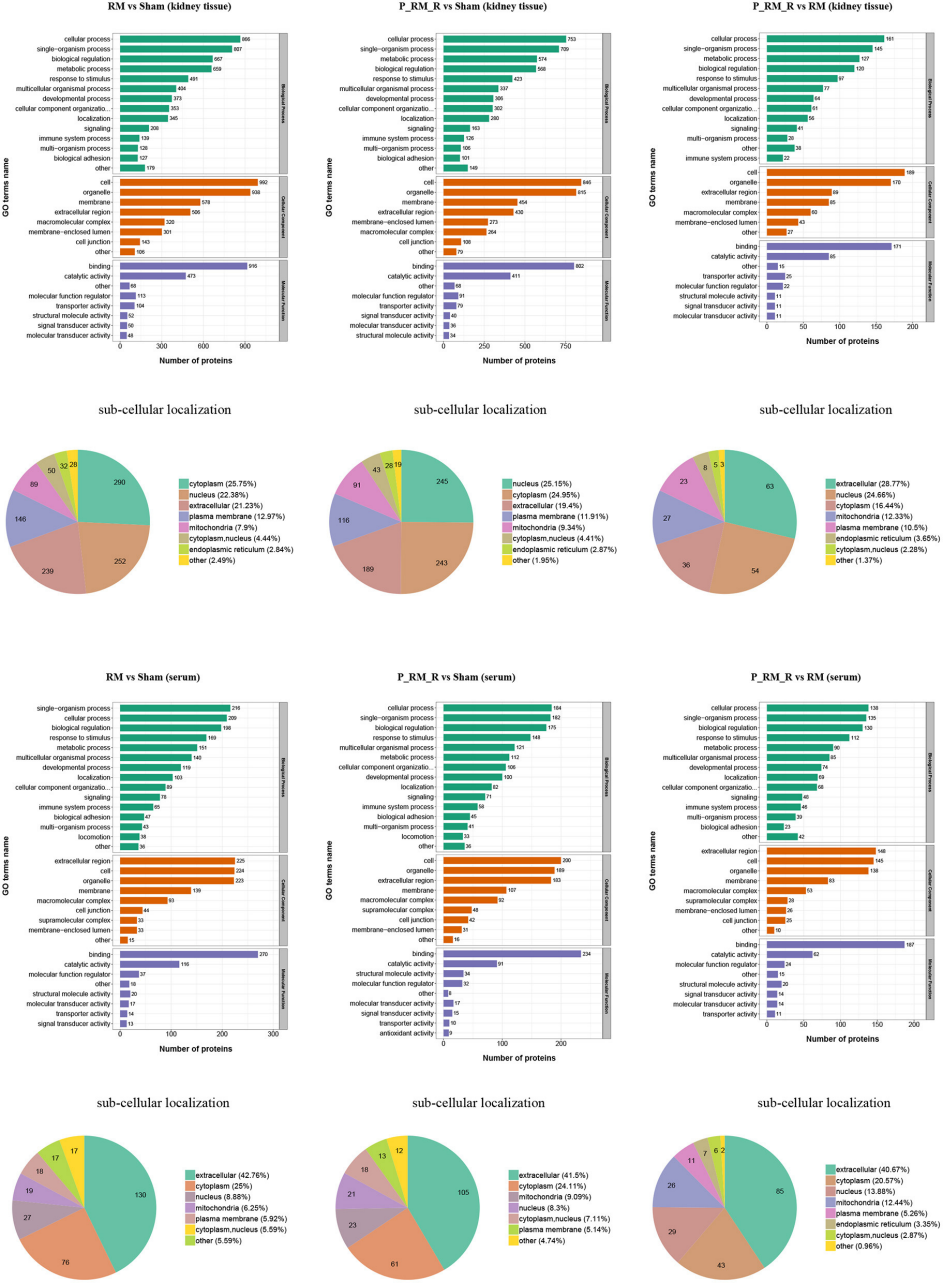
Gene Ontology terms and subcellular localization of proteins in kidney tissue and serum. RM, rhabdomyolysis; P_RM_R, the mouse in the parabiosis model administered glycerol; P_RM_S, the other mouse in the parabiosis model supplying the exogenous biological renal support.

**Figure 3:**
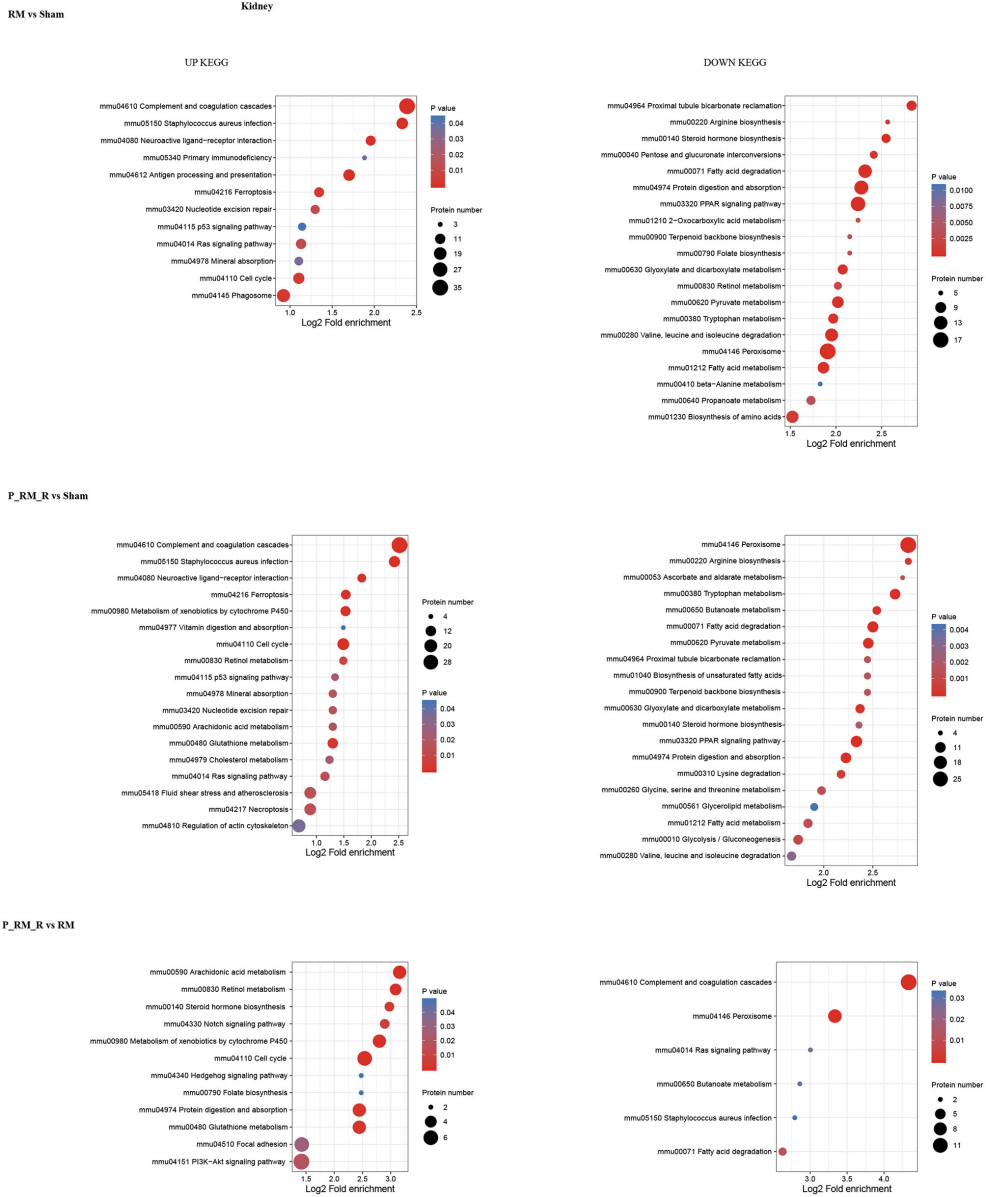
KEGG pathway enrichment of proteins in kidney tissue. RM, rhabdomyolysis; P_RM_R, the mouse in the parabiosis model administered glycerol; P_RM_S, the other mouse in the parabiosis model supplying the exogenous biological renal support.

**Figure 4:**
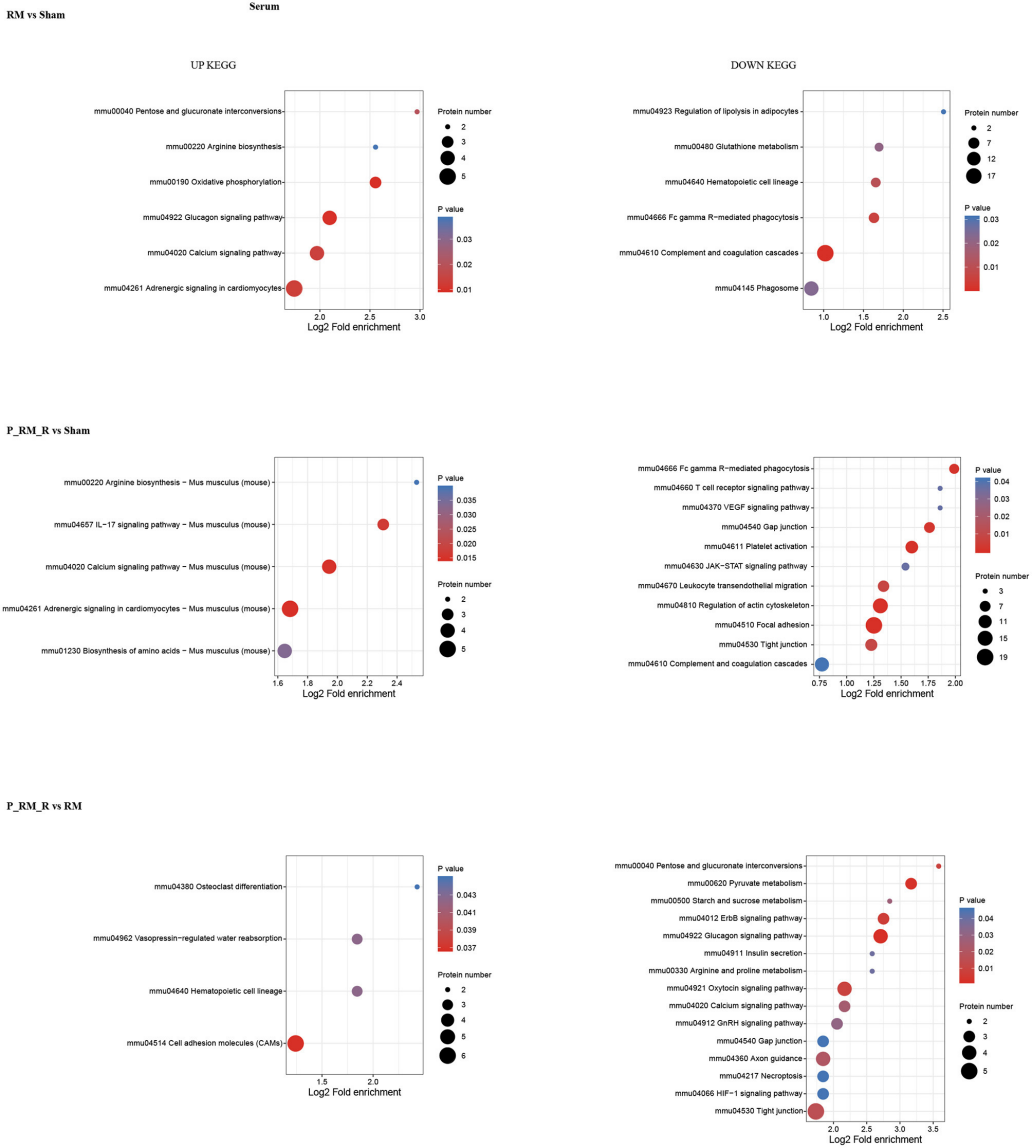
KEGG pathway enrichment of proteins in serum. RM, rhabdomyolysis; P_RM_R, the mouse in the parabiosis model administered glycerol; P_RM_S, the other mouse in the parabiosis model supplying the exogenous biological renal support.

To further understand the functions and features of the identified and quantified proteins, the proteins were classified into four categories based on their GO terms (Figure 2), subcellular localization (Figure 2), pathways (Figures 3, 4), and domains (Supplementary Figures 5, 6).

The number of differentially expressed proteins in each subcellular location was determined according to the subcellular location annotations of the identified proteins (Figure 2). Exogenous biological renal support led to increases in proteins associated with extracellular, nuclear, cytoplasmic, mitochondrial, and plasma membrane localization in kidney tissue in the parabiosis + RM group compared with the RM group. In addition, exogenous biological renal support induced increases in proteins associated with extracellular, cytoplasmic, nuclear, mitochondrial, and plasma membrane localization in serum in the parabiosis + RM group compared with the RM group (Figure 2).

Pathway functional enrichment analysis showed that exogenous biological renal support ameliorated AKI through multiple mechanisms, including by suppressing the complement system (mmu04610 complement and coagulation cascades), attenuating oxidative stress (mmu04146 peroxisome and mmu04014 Ras signaling pathway), ameliorating inflammation (mmu04020 calcium signaling pathway and mmu04610 complement and coagulation cascades), reducing apoptosis (mmu04020 calcium signaling pathway), alleviating necroptosis (mmu04217 necroptosis), and increasing proliferation (mmu04330 Notch signaling pathway, mmu04340 Hedgehog signaling pathway, mmmu04151 PI3K-Akt signaling pathway, and mmu04110 cell cycle).The top 30 upregulated and downregulated proteins are shown in Supplementary Table 1.

### Exogenous Biological Renal Support Suppressed Complement Activation in Mice With RM-Induced AKI

**Figure 5:**
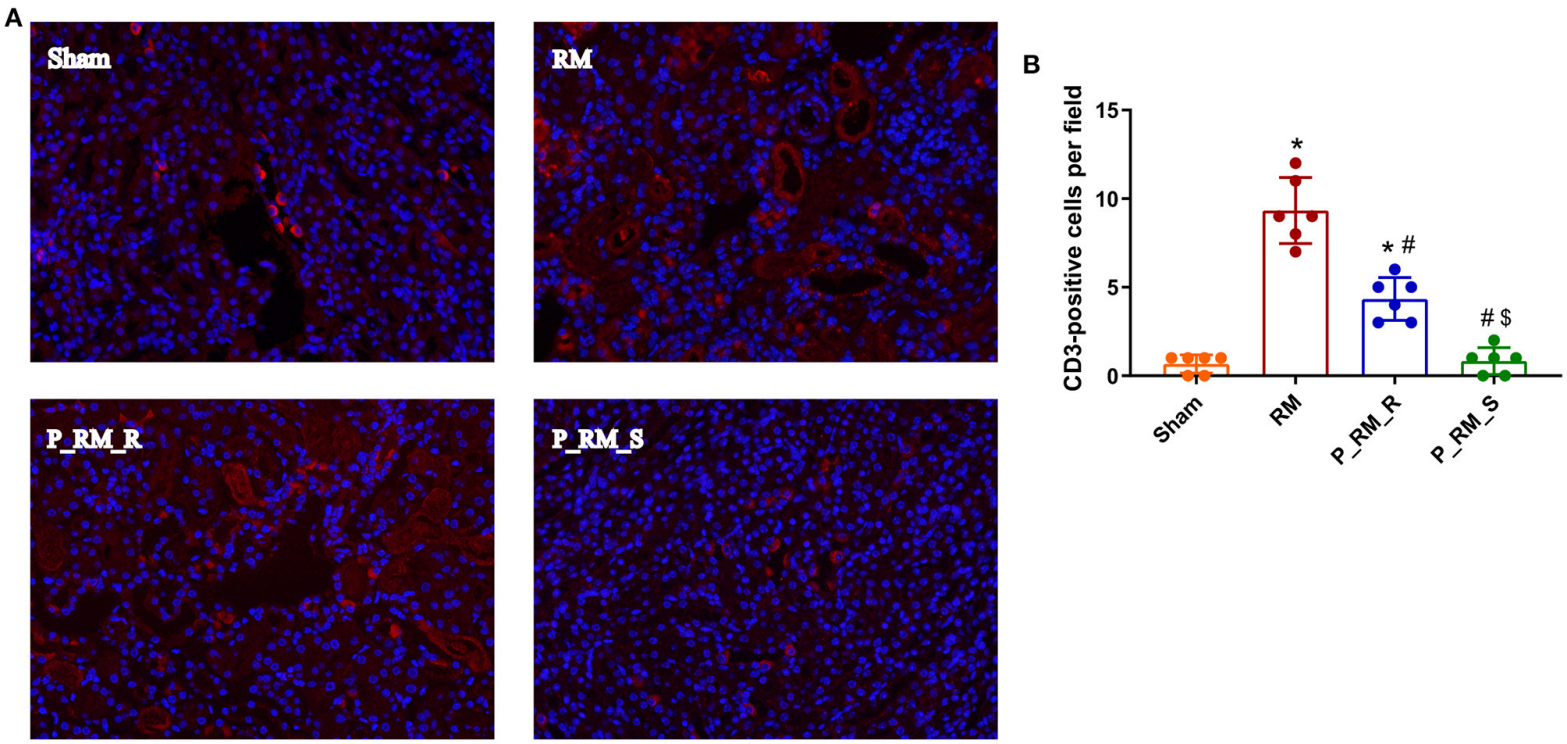
Immunofluorescence staining for C3. **(A)** Exogenous biological renal support decreased C3 deposition in RM-induced AKI. C3 was deposited along the renal tubular basement membrane in the RM and P_RM_R groups but not in the sham and P_RM_S groups. **(B)** Percentage of CD3-positive cells. RM, rhabdomyolysis; P_RM_R, the mouse in the parabiosis model administered glycerol; P_RM_S, the other mouse in the parabiosis model supplying the exogenous biological renal support. *P < 0.05 vs. the sham group; ^#^*P* < 0.05 vs. the RM group; ^$^*P* < 0.05 vs. the P_RM_R group.

Staining with the anti-C3 antibody revealed the expected weak signal in the parabiosis + RM group compared with the RM group at 48 h after RM-induced AKI in mice. These results indicate that complement system activation was suppressed by the exogenous biological renal support provided by parabiosis (Figure 5).

### Exogenous Biological Renal Support Decreased Oxidative Stress in Mice With RM-Induced AKI

**Figure 6:**
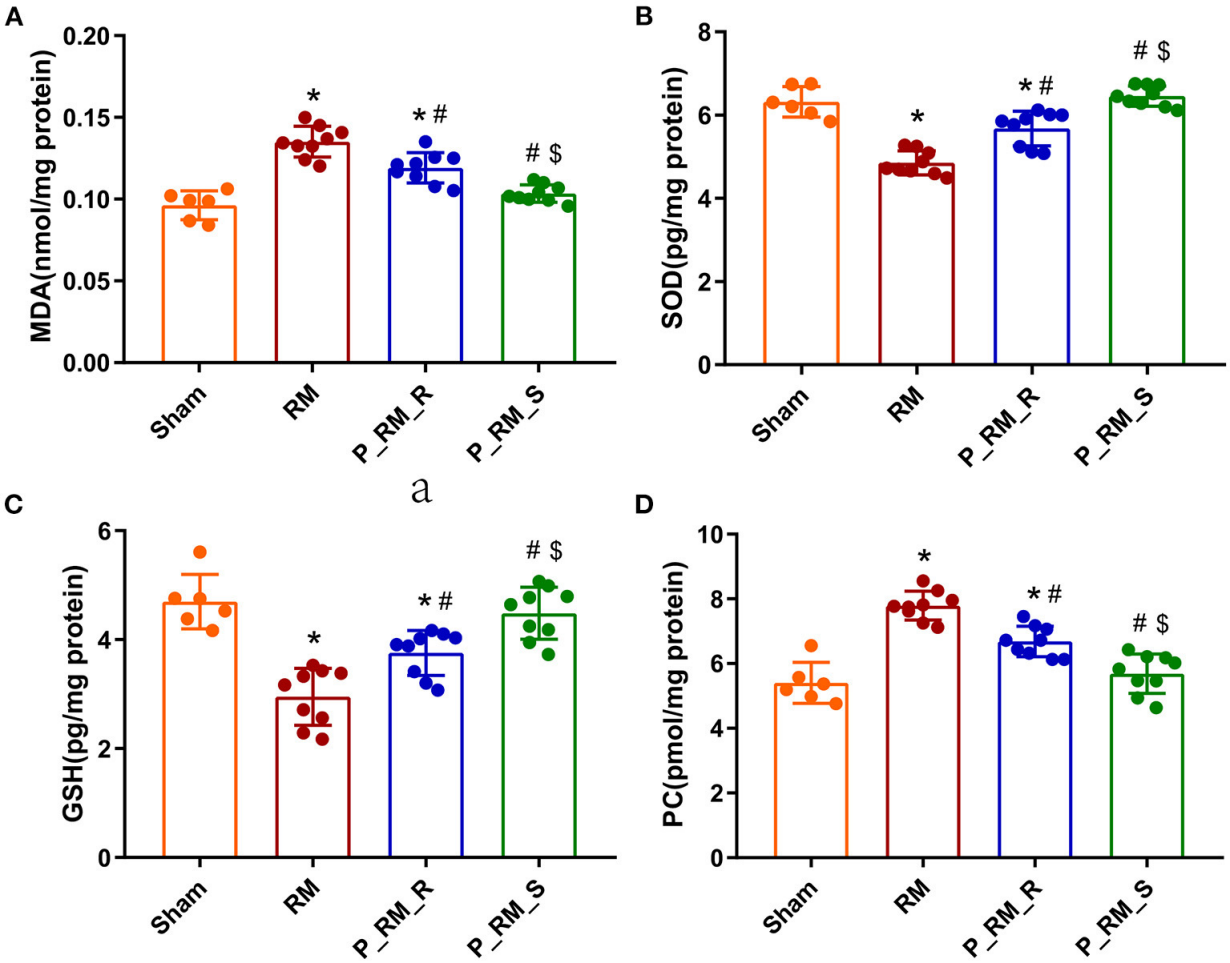
Exogenous biological renal support decreased oxidative stress in RM-induced AKI. **(A–D)** The levels of MDA, SOD, GSH, and PC were measured by ELISA. MDA, malondialdehyde; SOD, superoxide dismutase; GSH, glutathione peroxidase; PC, protein carbonyl; RM, rhabdomyolysis; P_RM_R, the mouse in the parabiosis model administered glycerol; P_RM_S, the other mouse in the parabiosis model supplying the exogenous biological renal support. *P < 0.05 vs. the sham group; ^#^*P* < 0.05 vs. the RM group; ^$^*P* < 0.05 vs. the P_RM_R group.

Compared with those in the RM group, the mice in the parabiosis + RM group showed higher SOD and GSH antioxidant enzymatic activity at 48 h after RM-induced AKI. In addition, the MDA and PC levels in the parabiosis + RM group were lower than those in the RM group. This finding indicates that there was less lipid and protein damage in the parabiosis + RM group (Figure 6).

### Exogenous Biological Renal Support Decreased Inflammation in Mice With RM-Induced AKI

**Figure 7:**
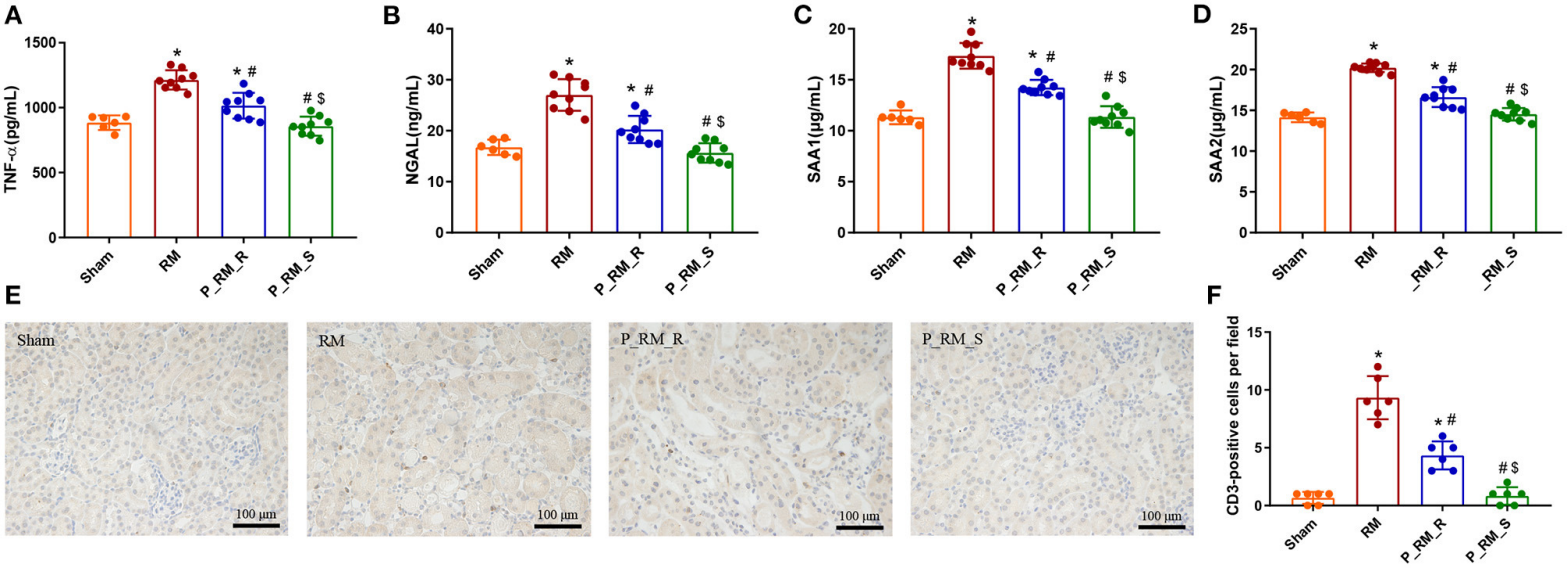
Exogenous biological renal support decreased inflammation in RM-induced AKI. **(A–D)** The levels of TNF-α, NGAL, SAA1, and SAA2 were measured by ELISA. **(E)** Representative images of renal CD3 expression in the four groups. **(F)** Comparison of the CD3-positive cells per field. TNF-α, tumor necrosis factor α; NGAL, neutrophil gelatinase-associated lipid carrier protein; SAA, serum amyloid A protein; RM, rhabdomyolysis; P_RM_R, the mouse in the parabiosis model administered glycerol; P_RM_S, the other mouse in the parabiosis model supplying the exogenous biological renal support. *P < 0.05 vs. the sham group; ^#^*P* < 0.05 vs. the RM group; ^$^*P* < 0.05 vs. the P_RM_R group.

The expression levels of TNF-α, SAA1, SAA2, and NGAL and the numbers of renal CD3-positive cells were significantly lower in the parabiosis + RM group than in the RM group. These results indicate that the renal inflammation level induced by RM may have been reduced by the exogenous biological renal support provided by the parabiosis model (Figure 7).

### Exogenous Biological Renal Support Decreased Apoptosis in Mice With RM-Induced AKI

**Figure 8:**
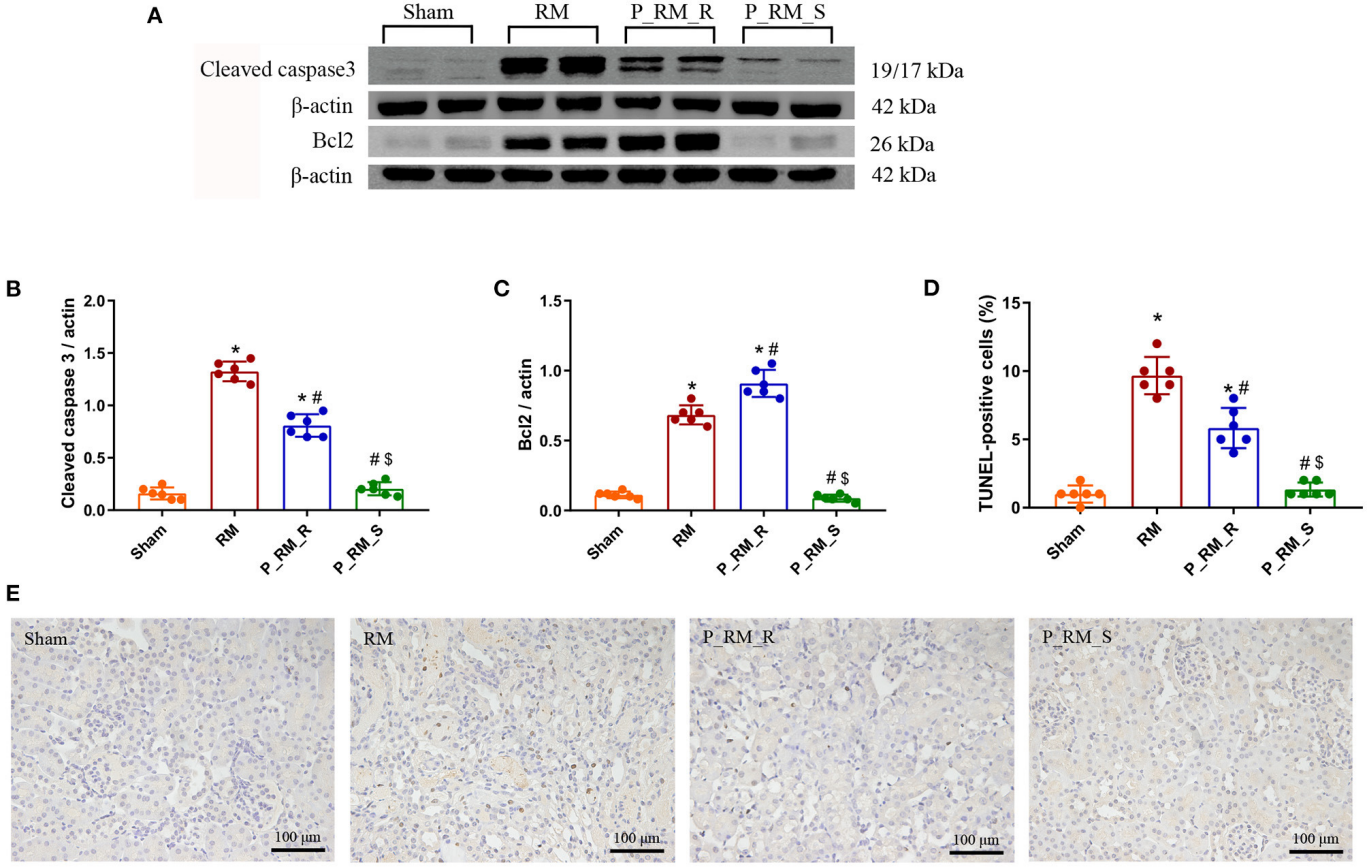
Exogenous biological renal support decreased apoptosis in RM-induced AKI. **(A–C)** The levels of Bcl2 and cleaved caspase-3 were measured by Western blot analysis. **(D)** Percentage of TUNEL-positive tubular cells. **(E)** TUNEL staining. RM, rhabdomyolysis; P_RM_R, the mouse in the parabiosis model administered glycerol; P_RM_S, the other mouse in the parabiosis model supplying the exogenous biological renal support. *P < 0.05 vs. the sham group; ^#^*P* < 0.05 vs. the RM group; ^$^*P* < 0.05 vs. the P_RM_R group.

The renal tissue expression levels of cleaved caspase-3 and the percentage of TUNEL-positive tubular cells were significantly lower in the parabiosis + RM group than in the RM group. The level of Bcl-2 was significantly higher in the parabiosis + RM group than in the RM group. These results indicate that the exogenous biological renal support provided by parabiosis may have alleviated apoptosis associated with RM-induced AKI (Figure 8).

### Exogenous Biological Renal Support Promoted Tubular Cell Proliferation in Mice With RM-Induced AKI

**Figure 9:**
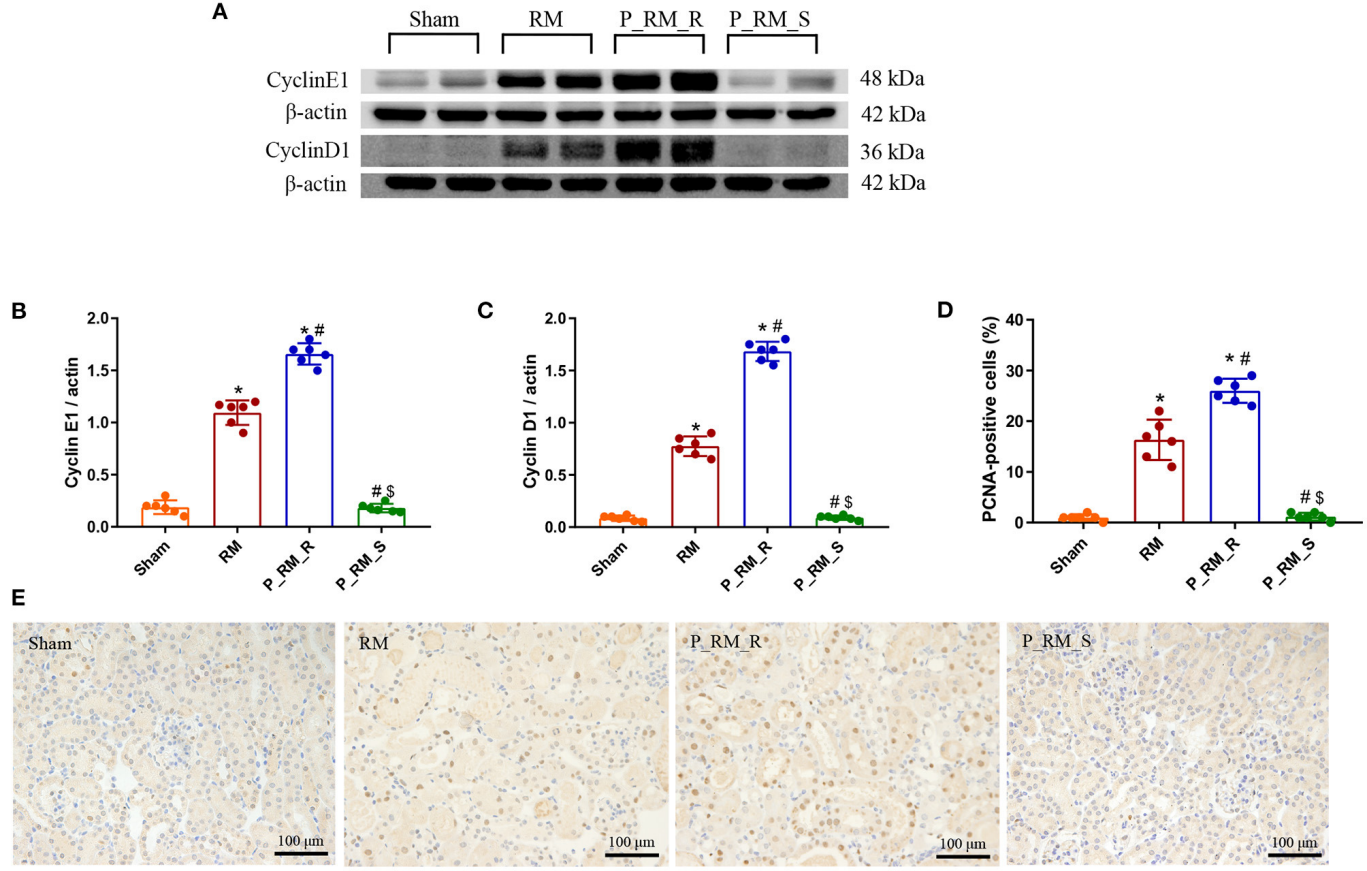
Exogenous biological renal support promotes tubular cell proliferation in RM-induced AKI. **(A)** The levels of cyclin D1 and cyclin E1 were measured by Western blot analysis. **(B,C)** Quantitative analyses of the band densities (protein expression) of cyclin D1 and cyclin E1. **(D)** Percentage of PCNA-positive tubular cells. **(E)** PCNA staining. RM, rhabdomyolysis; P_RM_R, the mouse in the parabiosis model administered glycerol; P_RM_S, the other mouse in the parabiosis model supplying the exogenous biological renal support. *P < 0.05 vs. the sham group; ^#^*P* < 0.05 vs. the RM group; ^$^*P* < 0.05 vs. the P_RM_R group.

At 48 h following RM-induced AKI in mice, the expression of cyclin D1 and cyclin E1 and the percentage of PCNA-positive cells were higher in the parabiosis + RM group than in the RM group. These findings indicate that the exogenous biological renal support provided by parabiosis significantly increased tubular cell proliferation in mice with RM-induced AKI (Figure 9).

## Discussion

In the parabiosis mouse model, the muscles and subcutaneous tissues of two mice are sutured together during surgery to form a shared blood circulatory system between the mice. Because of this shared blood circulatory system, blood cells and soluble factors are exchanged between the mice. Therefore, the use of parabiosis can provide exogenous biological renal support for AKI mice. Our previous research results have shown that exogenous biological renal support from young mice can reduce renal tissue inflammation, autophagy, and apoptosis and promote dedifferentiation and proliferation in aged mice after IRI model establishment (17, 24). We have also found that the expression of various inflammatory factors in renal tissue is upregulated in the aged mice, which may be related to the dedifferentiation, proliferation, and repair of damaged renal tissue cells (17, 27).

A previous study has demonstrated that, on average, 0.66% of the circulating blood volume is exchanged between parabiotic mice per hour. This exchange rate is equivalent to a mean daily exchange of 8% of the circulating volume (23). Our previous observational study has shown that GFP-positivse cells rarely enter the kidneys of wild-type mice that are conjoined with GFP mice (24). These observations suggest that the physiological benefits afforded to the mice with RM-induced AKI in the current study were unlikely to have resulted from exogenously transferred blood cells; rather, they were likely attributable to beneficial influences of the non-injured mice on water excretion, metabolism, transfer of bioactive molecules, and endocrine function in the injured mice.

The causes of RM include trauma, heat exposure, endurance running, drugs such as statins, infection, and hymenopteran stings (7). Animal models established using intramuscular glycerol injection with consequent myoglobinuria are closely related to the human syndrome of RM. RM-induced AKI is a major related adverse event and has been shown to be closely related to oxidative stress, renal inflammation, apoptosis, and proliferation (14, 27–29). In our study, the Scr and BUN levels in the RM group were significantly higher than those in the parabiosis + RM and sham groups. Sham mice did not show any significant tubular damage. RM mice showed tubular brush border loss, cast formation, tubular dilatation, and tubular necrosis accompanied by increased acute tubular injury scores. Parabiosis + RM mice showed significantly improved renal histological injury. To analyze the role of exogenous biological renal support, we used proteomic analysis to identify key proteins and key pathways. The results showed that compared with the mice in the RM group, the mice with exogenous biological renal support exhibited increases in proteins associated with extracellular, nuclear, cytoplasmic, mitochondrial, and plasma membrane localization in kidney tissue and serum. Pathway functional enrichment analysis identified suppression of complement system activation; attenuation of oxidative stress, inflammation, and cell death; and increased proliferation. We further confirmed these effects with *in vivo* experiments.

Tubular changes are usually sublethal and develop within 24 h after the onset of RM (30, 31). By 72 h, glycerol-treated mice develop AKI and exhibit significantly increased Scr and BUN levels and significant morphological changes (26). In this study, to observe the effect of exogenous biological renal support, we collected specimens at 48 h after glycerol injection.

MYO and CK are the main products of the breakdown of skeletal muscle fibers in glycerol-induced RM models (32). MYO-induced renal toxicity plays a key role in RM-induced AKI by activating the complement system and increasing oxidative stress, inflammation, endothelial dysfunction, vasoconstriction, and cell death (11–13). The characteristic clinical symptoms of RM are elevated serum CK and MYO levels (1). Our results showed that the levels of CK and MYO were not significantly different between the RM and P_RM_R groups (Supplementary Figure 7). Possible reasons are as follows: on the one hand, the blood exchange rate between parabiotic mice is not high (only 8% is exchanged each day), and the amount of blood exchanged at 48 h after RM is established is limited; on the other hand, previous research has indicated that a number of biological systems are activated following muscle extract infusion and that these systems may be more important than the nephrotoxicity of MYO in the pathogenesis of renal injury (33). In addition, a recent study performed on critically ill patients showed that although MYO clearance was higher in the intervention arm (subjected to continuous venovenous hemodialysis with a high-cutoff dialyzer) than in the control arm (subjected to continuous venovenous hemodiafiltration), there were no differences in hospital mortality, 28- or 90-day mortality, adverse events, or severe adverse events between the groups (34). Our research results show that the effects of exogenous biological renal support are not entirely attributable to MYO clearance, but that this support strategy can alleviate AKI by suppressing complement system activation; decreasing oxidative stress, inflammation, and cell death; and promoting tubular cell proliferation.

The proteomic analysis results show that both apoptosis and necroptosis are activated during renal cell death in RM-induced AKI. Ferroptosis (mmu04216), the most important cell death pathway involved in RM-induced AKI (35, 36), was upregulated in the RM group vs. the sham group and in the P_RM_R group vs. the sham group but was not significantly regulated in the P_RM_R group vs. the RM group. Whether exogenous biological renal support can effectively regulate the ferroptosis pathway needs further assessment via bioinformatics analysis and verification *in vivo* and *in vitro*. In addition, the proteomic analysis results show that metabolic signaling pathways are involved in this process and that metabolic reprogramming may be an important part of kidney regeneration and repair. Therefore, additional study is needed to explore the mechanism. Furthermore, exogenous biological renal support has multifaceted effects on the recovery of renal function, and the effects of key pathways or proteins cannot be independently verified with parabiosis models; these effects should be further examined in single mouse models of RM.

## Conclusion

In summary, we have demonstrated that exogenous biological renal support supplied by parabiosis can improve renal function in RM-induced AKI by suppressing complement system activation; decreasing oxidative stress, inflammation, and cell death; and promoting tubular cell proliferation. Our study provides new ideas for effectively preventing and treating RM-induced AKI and provides basic research evidence for the use of bioartificial kidneys to treat RM-induced AKI.

## Data Availability Statement

The original contributions presented in the study are included in the article/Supplementary Material, further inquiries can be directed to the corresponding author/s.

## Ethics Statement

The animal study was reviewed and approved by Animal Ethics Committee of the Chinese PLA General Hospital.

## Author Contributions

CL, QHo, XC, and XS designed the research. CL, KC, XG, ZM, QHo, DL, YW, and YZ performed the research. QHu, ZM, FZ, GC, and XS methodology. CL, KC, XG, ZM, QHu, GC, XC, and FZ analyzed the data. CL, KC, XG, ZM, and XS wrote the manuscript. All authors revised the manuscript draft and approved the final version for submission.

## Conflict of Interest

The authors declare that the research was conducted in the absence of any commercial or financial relationships that could be construed as a potential conflict of interest.

## Supplementary Material

Supplementary Figure 1 Parabiosis procedure. **(A)** Anesthesia and skin preparation. Semicircular markings were made on the lateral abdomens of both the donor and recipient. **(B,C)** The skin and subcutaneous tissue were separated, and then the marked skin was cut to expose the subcutaneous muscle. **(D–F)** The chest muscle flap of the donor mouse was isolated and then sutured to the chest of the recipient mouse. The abdominal muscles of the two mice were connected with 3-point interrupted sutures. **(G,H)** The outer skin of the two mice was sutured. Successful surgery relied on the establishment of a shared blood circulation, which required a minimum 1- to 1.5-cm^2^ area of isolated chest and abdomen muscle flap.

Supplementary Figure 2 Confirmation of shared blood circulation after parabiosis. (1) Peripheral blood smear test for GFP detection: established isochronic (3 months) parabiosis of C57BL/6 GFP mouse (donor) and C57BL/6 wild-type mouse (receptor); three weeks after parabiosis, venous blood from the donor and recipient were taken for the smear test; and the area of green fluorescent protein was observed under a fluorescent microscope. **(A–D)** Fluorescence microscopy of peripheral blood smears. **(A)** No green fluorescent protein (GFP) positive blood cells were observed in the peripheral blood smear from the wild-type mouse. **(B)** GFP-positive blood cells in the peripheral blood smear from the enhanced GFP (EGFP) transgenic mouse. **(C,D)** Three weeks after parabiosis was established between the EGFP transgenic mouse and the wild-type mouse, GFP-positive blood cells were observed in the peripheralblood smear of the **(C)** EGFP transgenic mouse and the **(D)** wild-type mouse. (2) Flow cytometry for the measurement of GFP+ cell ratio: established the same animal model as (1), prepared leukocyte blood cells from the venous blood of the donor and recipient, and the ratio of GFP+ cells in the red blood cells of donor and recipient mice was measured by flow cytometry (CYTOMICS FC 500; Beckman Coulter Inc., USA), FlowJo Software, version 7.6 (Tree Star Inc., Ashland, OR), was used for data analysis. **(E–H)** Flow cytometry measurements. **(E)** The wild-type mice had a negligible amount of GFP-positive cells in the peripheral blood. **(F)** The majority of peripheral blood cells in the EGFP mice were GFP positive. In the parabiosis model, the amounts of GFP-positive cells in the peripheral blood of the **(G)** EGFP transgenic mouse and **(H)** wild-type mouse were similar.(3) Small animal *in vivo* imaging: established isochronic (3 months) parabiosis of C57BL/6 wild-type mouse (donor) and C57BL/6 wild-type mouse (receptor). Two weeks after the parabiosis, DiR (100 μl, 16 μl/ml of PBS) were injected into the caudal vein of the donor mouse. Thirty minutes after injection, the distribution of DiR in the donor and recipient mice was traced using a small animal in vivo imaging system (IVIS Spectrum, US Caliper Life Sciences). **(I–N)** Using a small animal in vivo imaging system, we observed the body surface positions of fluorescent dyes in mice. **(I)** Wild-type mouse injected with phosphate-buffered saline. **(G)** Wild-type mouse injected with 1,1‘-dioctadecyl-3,3,3‘,3‘-tetramethylindotricarbocyanine iodide (DiR) fluorescent dye. **(I,K)** Parabiosis model. **(K)** After 30 min to 2 h, the distribution of DiR was observed at the spleen's body surface position in the other mouse. **(I)** DiR fluorescent dye was injected into 1 mouse of the parabiosis model. **(M,N)** Parabiosis model. **(M)** After 30 min to 2 h, the distribution of DiR was observed at the liver's body surface position in the other mouse. **(N)** DiR fluorescent dye was injected into 1 mouse of the parabiosis model.

Supplementary Figure 3 Principal component analysis.

Supplementary Figure 4 Differentially expressed protein statistics.

Supplementary Figure 5 Domain enrichment analysis of proteins in kidney tissue.

Supplementary Figure 6 Domain enrichment analysis of proteins in serum.

Supplementary Figure 7 CK and myoglobin (MYO) expression levels.

Supplementary Table 1 Different proteins in the P_RM_R group vs. the RM group at 48 h. RM, rhabdomyolysis; P_RM_R, the mouse in the parabiosis model administered glycerol.

## Abbreviations

AKI: acute kidney injury
GO: Gene Ontology
KEGG: Kyoto Encyclopedia of Genes and Genomes
RM: rhabdomyolysis
P_RM_R: the mouse in the parabiosis model administered glycerol
P_RM_S: the other mouse in the parabiosis model that supplied the exogenous biological renal support.
