# Supplementary figures and images for: Exogenous Biological Renal Support Improves Kidney Function in Mice With Rhabdomyolysis-Induced Acute Kidney Injury
Source: Front Med (Lausanne). 2021 May 28;8:655787. doi: 10.3389/fmed.2021.655787 (PMC8193099; doi:10.3389/fmed.2021.655787)

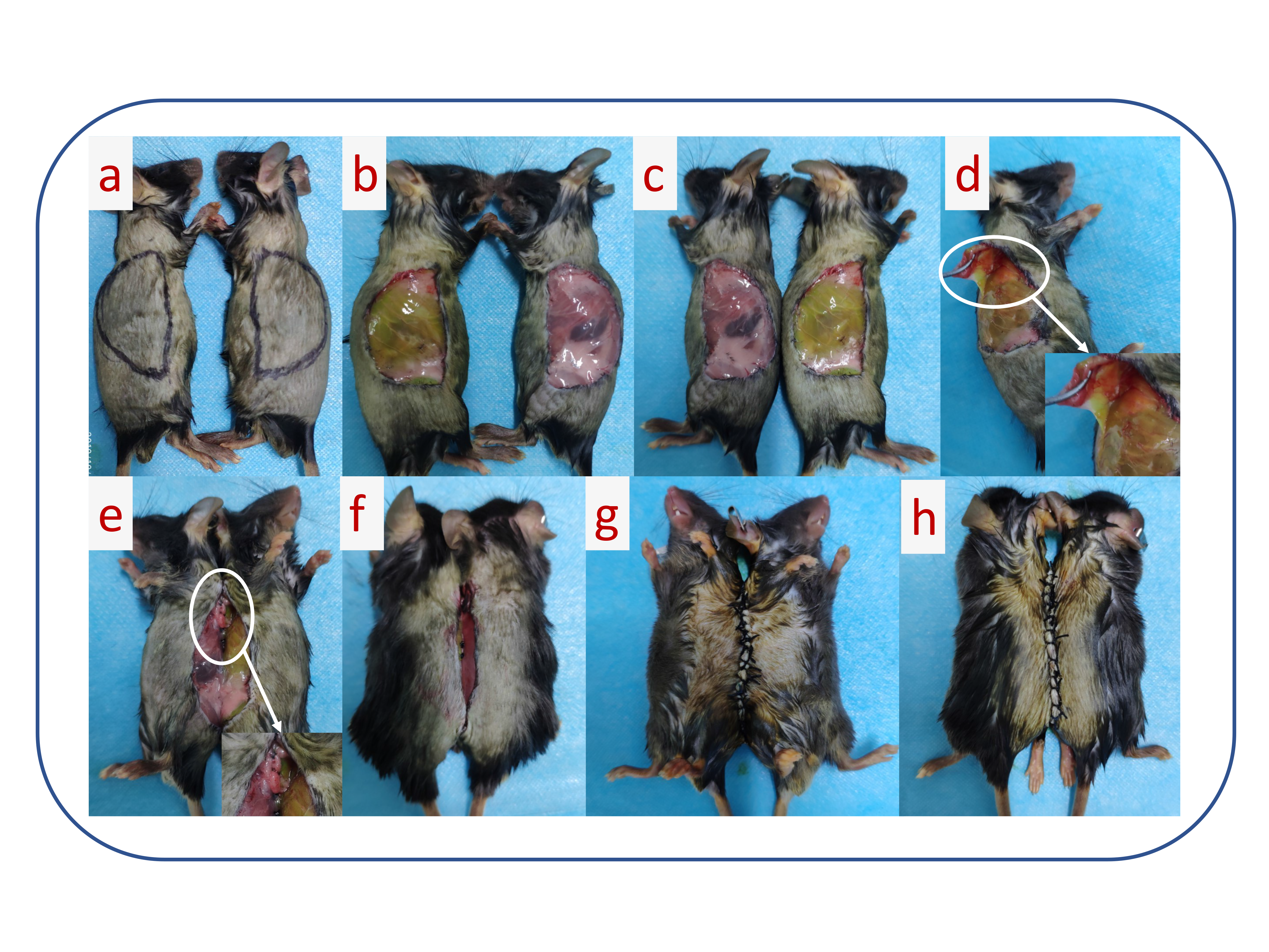

Supplement: Supplementary Figure 1 — Parabiosis procedure. (A) Anesthesia and skin preparation. Semicircular markings were made on the lateral abdomens of both the donor and recipient. (B,C) The skin and subcutaneous tissue were separated, and then the marked skin was cut to expose the subcutaneous muscle. (D–F) The chest muscle flap of the donor mouse was isolated and then sutured to the chest of the recipient mouse. The abdominal muscles of the two mice were connected with 3-point interrupted sutures. (G,H) The outer skin of the two mice was sutured. Successful surgery relied on the establishment of a shared blood circulation, which required a minimum 1- to 1.5-cm2 area of isolated chest and abdomen muscle flap. [file Image_1.TIF]

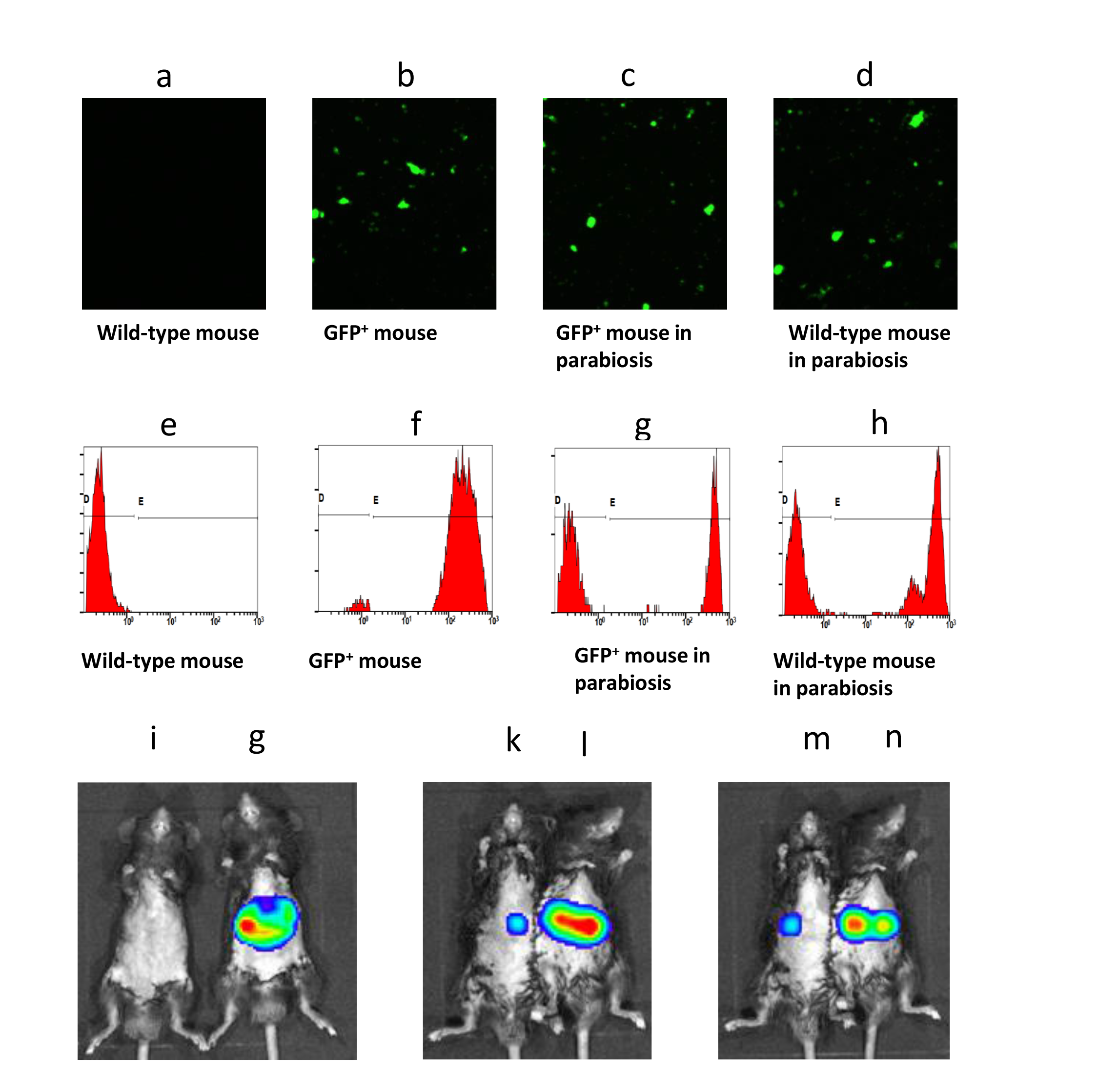

Supplement: Supplementary Figure 2 — Confirmation of shared blood circulation after parabiosis. (1) Peripheral blood smear test for GFP detection: established isochronic (3 months) parabiosis of C57BL/6 GFP mouse (donor) and C57BL/6 wild-type mouse (receptor); three weeks after parabiosis, venous blood from the donor and recipient were taken for the smear test; and the area of green fluorescent protein was observed under a fluorescent microscope. (A–D) Fluorescence microscopy of peripheral blood smears. (A) No green fluorescent protein (GFP) positive blood cells were observed in the peripheral blood smear from the wild-type mouse. (B) GFP-positive blood cells in the peripheral blood smear from the enhanced GFP (EGFP) transgenic mouse. (C,D) Three weeks after parabiosis was established between the EGFP transgenic mouse and the wild-type mouse, GFP-positive blood cells were observed in the peripheralblood smear of the (C) EGFP transgenic mouse and the (D) wild-type mouse. (2) Flow cytometry for the measurement of GFP+ cell ratio: established the same animal model as (1), prepared leukocyte blood cells from the venous blood of the donor and recipient, and the ratio of GFP+ cells in the red blood cells of donor and recipient mice was measured by flow cytometry (CYTOMICS FC 500; Beckman Coulter Inc., USA), FlowJo Software, version 7.6 (Tree Star Inc., Ashland, OR), was used for data analysis. (E–H) Flow cytometry measurements. (E) The wild-type mice had a negligible amount of GFP-positive cells in the peripheral blood. (F) The majority of peripheral blood cells in the EGFP mice were GFP positive. In the parabiosis model, the amounts of GFP-positive cells in the peripheral blood of the (G) EGFP transgenic mouse and (H) wild-type mouse were similar.(3) Small animal in vivo imaging: established isochronic (3 months) parabiosis of C57BL/6 wild-type mouse (donor) and C57BL/6 wild-type mouse (receptor). Two weeks after the parabiosis, DiR (100 μl, 16 μl/ml of PBS) were injected into the caudal vein of the donor m [file Image_2.TIF]

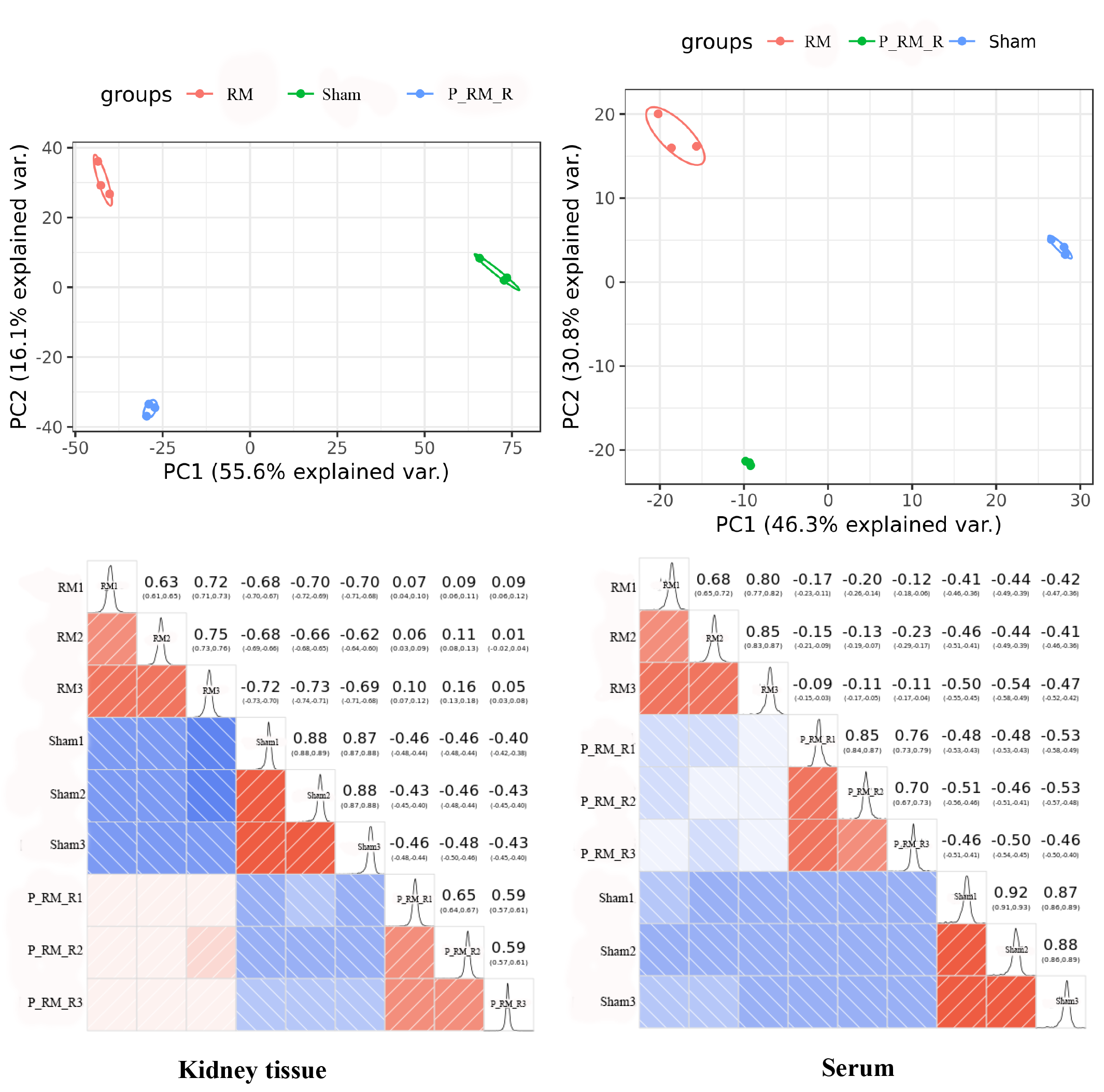

Supplement: Supplementary Figure 3 — Principal component analysis. [file Image_3.TIF]

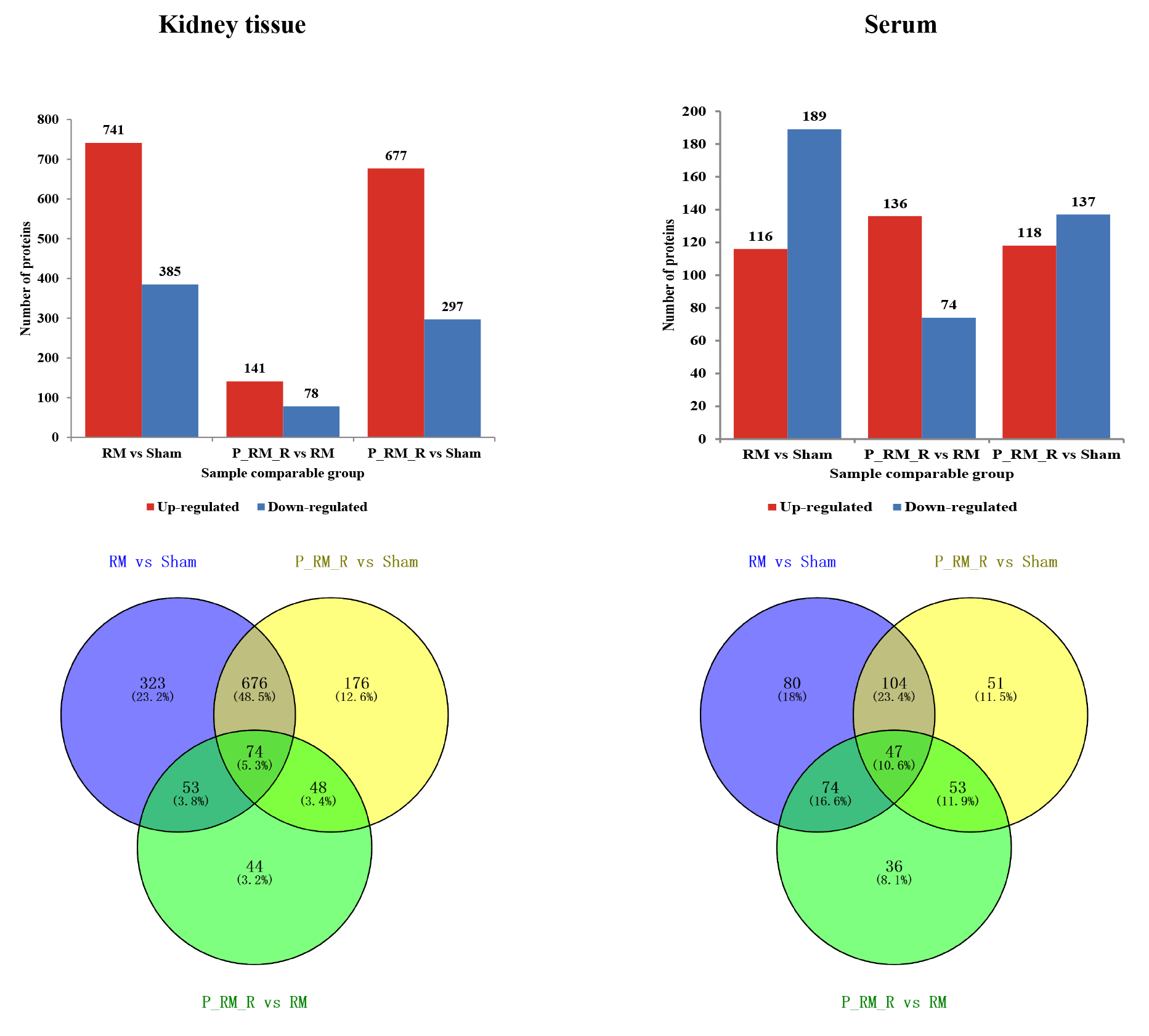

Supplement: Supplementary Figure 4 — Differentially expressed protein statistics. [file Image_4.TIF]

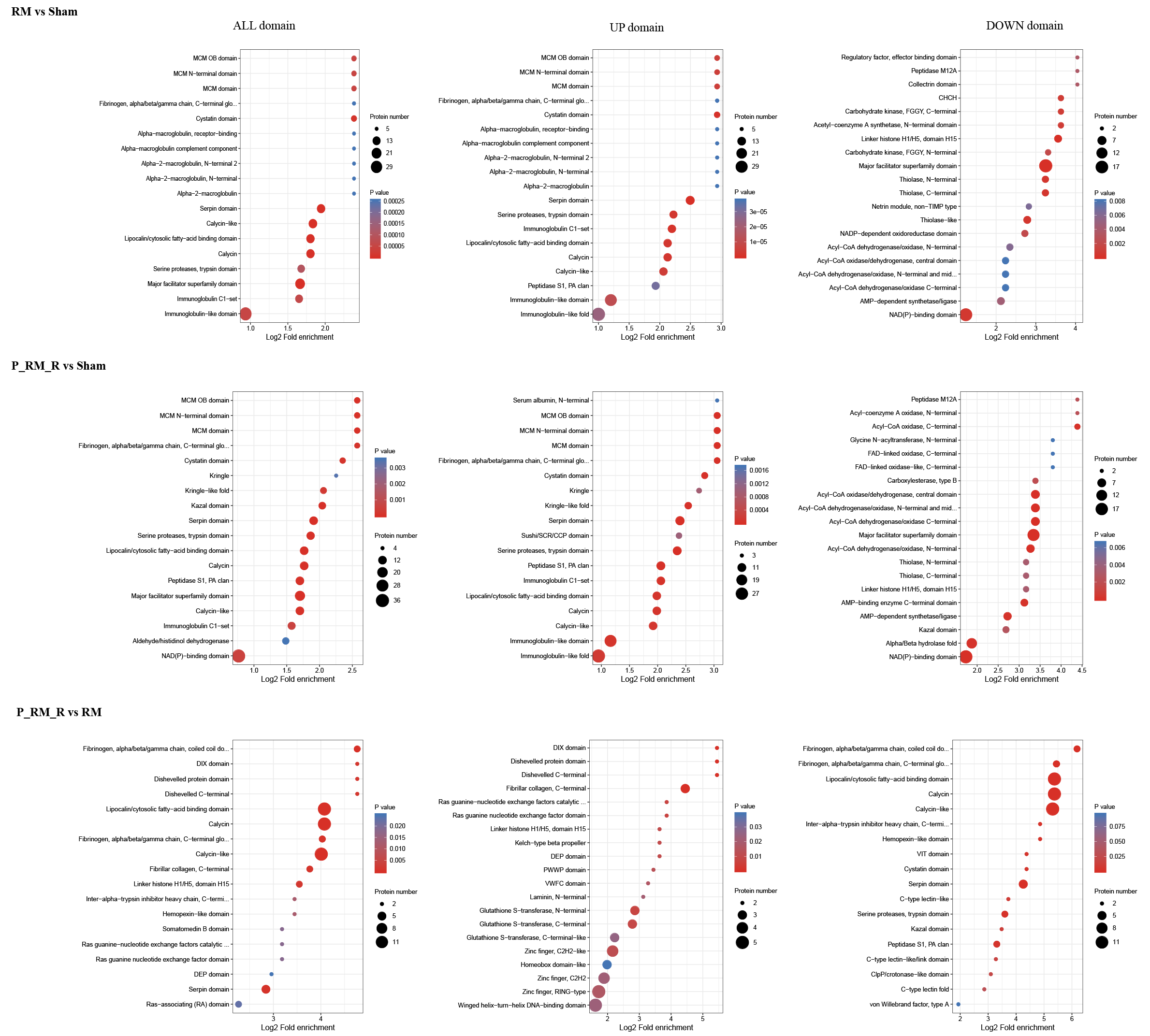

Supplement: Supplementary Figure 5 — Domain enrichment analysis of proteins in kidney tissue. [file Image_5.TIF]

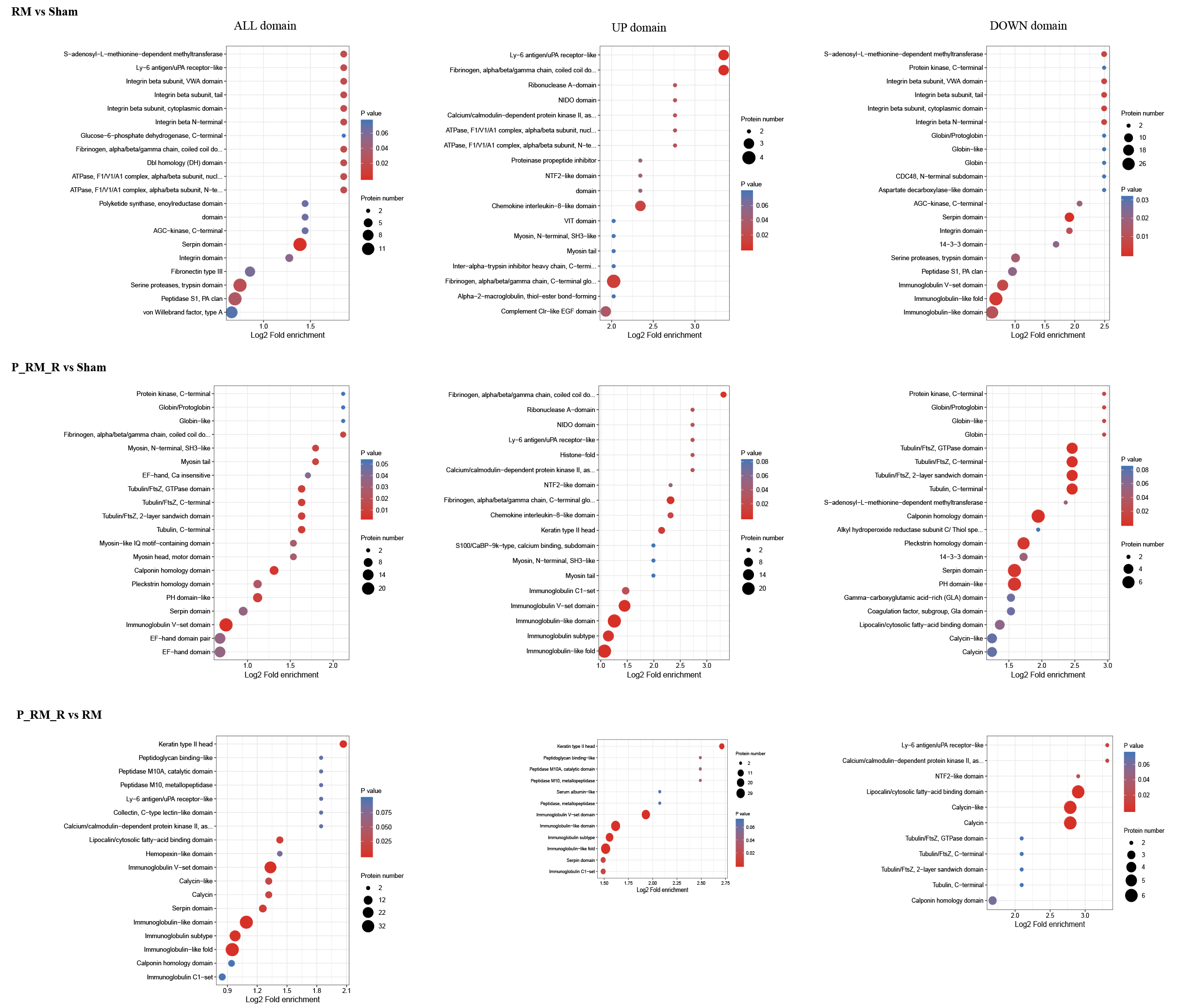

Supplement: Supplementary Figure 6 — Domain enrichment analysis of proteins in serum. [file Image_6.TIF]

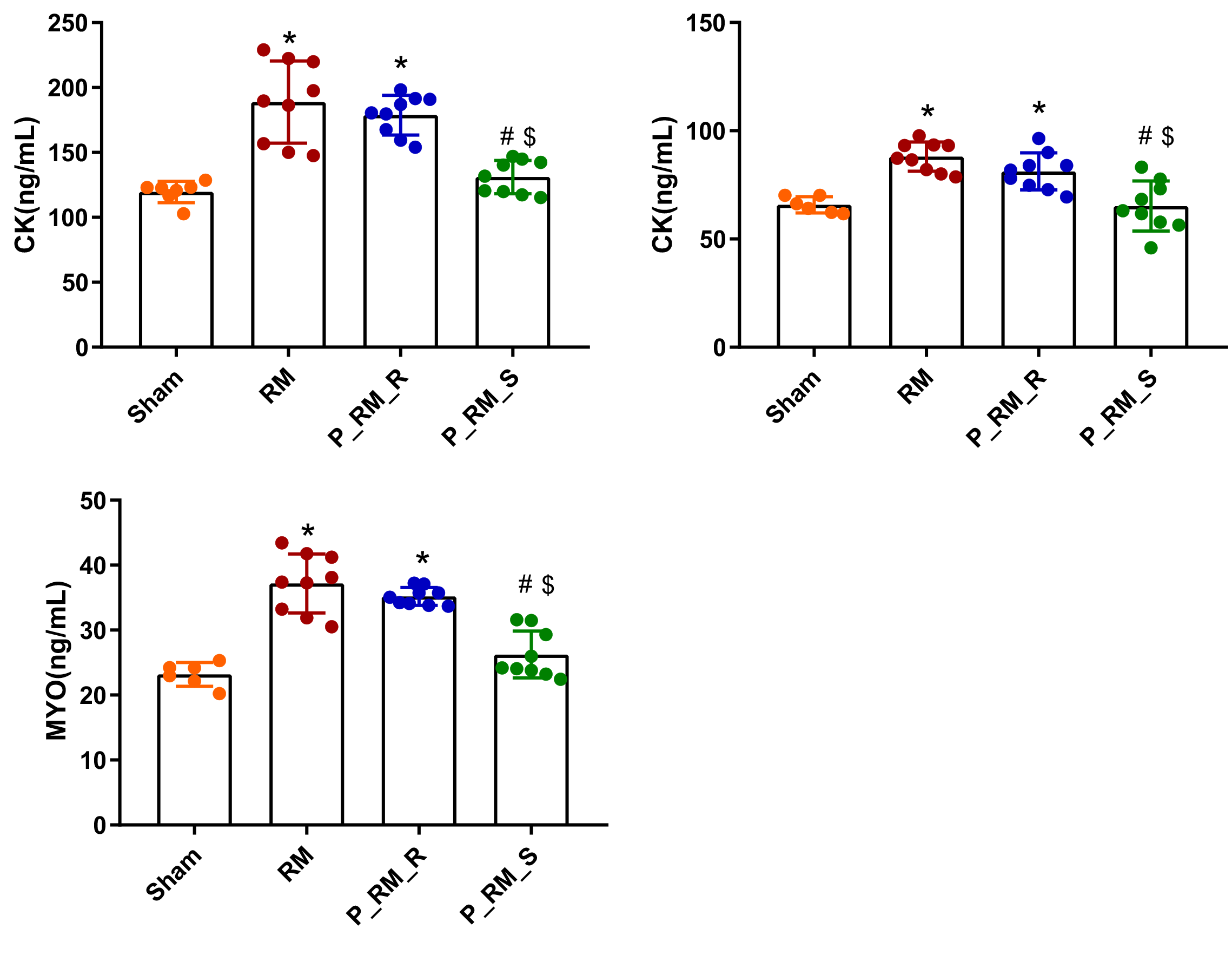

Supplement: Supplementary Figure 7 — CK and myoglobin (MYO) expression levels. [file Image_7.TIF]
