# Supplementary material for: Exogenous Biological Renal Support Improves Kidney Function in Mice With Rhabdomyolysis-Induced Acute Kidney Injury
Source: Front Med (Lausanne). 2021 May 28;8:655787. doi: 10.3389/fmed.2021.655787 (PMC8193099; doi:10.3389/fmed.2021.655787)
Supplement: Supplementary Table 1 — Different proteins in the P_RM_R group vs. the RM group at 48 h. RM, rhabdomyolysis; P_RM_R, the mouse in the parabiosis model administered glycerol. [file Data_Sheet_1.docx]

| P_RM_R versus RM at 48h (p ＜0.05). | | | | | | | |
| --- | --- | --- | --- | --- | --- | --- | --- |
| **Top 30 upregulated** | | | | | | | |
| **Kidney tissues specimen** | | | | **Serum specimen** | | | |
| **Protein accession** | **Gene name** | **P_RM_R/ RM Ratio** | **Subcellular localization** | **Protein accession** | **Gene name** | **P_RM_R/ RM Ratio** | **Subcellular localization** |
| Q810C0 | Slitrk2 | 2.996 | plasma membrane | Q9CXT8 | Pmpcb | 5.389 | mitochondria |
| O35728 | Cyp4a14 | 2.286 | plasma membrane | P31725 | S100a9 | 4.303 | cytoplasm |
| Q9CXF0 | Kynu | 2.189 | cytoplasm | P01868 | Ighg1 | 3.254 | extracellular |
| P01868 | Ighg1 | 2.13 | extracellular | Q8C1F5 | Ttc16 | 2.771 | nucleus |
| Q99P87 | Retn | 1.83 | extracellular | P06327 | Gm5629 | 2.595 | extracellular |
| A2AEP0 | Obp1b | 1.815 | extracellular | Q9DCH4 | Eif3f | 2.363 | mitochondria |
| A2ATU0 | Dhtkd1 | 1.781 | mitochondria | P14733 | Lmnb1 | 2.278 | nucleus |
| O70451 | Slc16a7 | 1.779 | plasma membrane | Q62087 | Pon3 | 2.205 | extracellular |
| Q07409 | Cntn3 | 1.765 | extracellular | Q8BQN6 | Znf146 | 2.194 | nucleus |
| P10648 | Gsta2 | 1.755 | cytoplasm | Q60610 | Tiam1 | 2.177 | nucleus |
| Q9D952 | Evpl | 1.748 | nucleus | O08692 | Ngp | 2.173 | extracellular |
| Q925P2 | Ceacam2 | 1.693 | endoplasmic reticulum | P32261 | Serpinc1 | 2.155 | endoplasmic reticulum |
| P56393 | Cox7b | 1.688 | mitochondria | Q8K426 | Retnlg | 2.126 | extracellular |
| Q61062 | Dvl3 | 1.688 | nucleus | Q6PA06 | Atl2 | 2.052 | plasma membrane |
| Q8CGA3 | Slc43a2 | 1.683 | plasma membrane | P06328 | Ighv1-72 | 1.987 | extracellular |
| Q08091 | Cnn1 | 1.67 | cytoplasm | Q5U405 | Tmprss13 | 1.96 | nucleus |
| O35744 | Chil3 | 1.665 | extracellular | P43277 | Hist1h1d | 1.951 | nucleus |
| P17918 | Pcna | 1.634 | cytoplasm | P27005 | S100a8 | 1.896 | cytoplasm |
| Q64373 | Bcl2l1 | 1.629 | extracellular | P11247 | Mpo | 1.88 | endoplasmic reticulum |
| Q8BI29 | Sarg | 1.606 | nucleus | Q8VED5 | Krt79 | 1.878 | nucleus |
| Q9NYQ2 | Hao2 | 1.595 | cytoplasm | Q8R4Y4 | Stab1 | 1.827 | plasma membrane |
| P24549 | Aldh1a1 | 1.594 | cytoplasm | Q45VN2 | Defa20 | 1.789 | extracellular |
| O35945 | Aldh1a7 | 1.566 | cytoplasm | Q9JI91 | Actn2 | 1.77 | cytoplasm,nucleus |
| Q9JI33 | Ntn4 | 1.566 | extracellular | O09164 | Sod3 | 1.769 | extracellular |
| P25322 | Ccnd1 | 1.565 | nucleus | P08071 | Ltf | 1.766 | endoplasmic reticulum |
| Q04735 | Cdk16 | 1.559 | nucleus | Q61233 | Lcp1 | 1.746 | nucleus |
| Q62445 | Sp4 | 1.554 | nucleus | A6BLY7 | Krt28 | 1.715 | plasma membrane |
| Q8K4S1 | Plce1 | 1.548 | nucleus | P51437 | Camp | 1.715 | extracellular |
| Q8C6K9 | Col6a6 | 1.547 | endoplasmic reticulum | P49182 | Serpind1 | 1.709 | cytoplasm,nucleus |
| Q91Z31 | Ptbp2 | 1.544 | cytoplasm | Q08048 | Hgf | 1.692 | extracellular |
| **Top 30 downregulated** | | | | | | | |
| **Kidney tissues specimen** | | | | **Serum specimen** | | | |
| **Protein accession** | **Gene name** | **P_RM_R/ RM Ratio** | **Subcellular localization** | **Protein accession** | **Gene name** | **P_RM_R/ RM Ratio** | **Subcellular localization** |
| P11588 | Mup1 | 0.169 | extracellular | P12246 | Apcs | 0.424 | extracellular |
| B5X0G2 | Mup17 | 0.239 | extracellular | P11588 | Mup1 | 0.455 | extracellular |
| Q80T69 | Rsbn1 | 0.246 | nucleus | P16125 | Ldhb | 0.461 | cytoplasm |
| P35230 | Reg3b | 0.35 | extracellular | P11589 | Mup2 | 0.479 | extracellular |
| P11589 | Mup2 | 0.361 | extracellular | Q91XE4 | Acy3 | 0.501 | extracellular |
| Q8BH70 | Fbxl4 | 0.373 | mitochondria | Q91ZJ5 | Ugp2 | 0.505 | cytoplasm |
| P04939 | Mup3 | 0.403 | extracellular | P16460 | Ass1 | 0.511 | cytoplasm |
| O09049 | Reg3g | 0.415 | extracellular | Q8BYW1 | Arhgap25 | 0.514 | nucleus |
| P12246 | Apcs | 0.417 | extracellular | P63038 | Hspd1 | 0.52 | mitochondria |
| Q00898 | Serpina1e | 0.447 | extracellular | P05367 | Saa2 | 0.55 | extracellular |
| P34928 | Apoc1 | 0.475 | extracellular | P05366 | Saa1 | 0.561 | extracellular |
| P07361 | Orm2 | 0.507 | extracellular | Q9JM99 | Prg4 | 0.563 | extracellular |
| P59913 | Pcmtd1 | 0.51 | cytoplasm,nucleus | Q61696 | Hspa1a | 0.568 | cytoplasm |
| P11591 | Mup5 | 0.511 | extracellular | P28666 | Mug2 | 0.588 | extracellular |
| Q61646 | Hp | 0.511 | extracellular | P11672 | Lcn2 | 0.593 | extracellular |
| Q60590 | Orm1 | 0.529 | extracellular | O88338 | Cdh16 | 0.601 | extracellular |
| P29788 | Vtn | 0.535 | extracellular | P56480 | Atp5f1b | 0.603 | mitochondria |
| P70677 | Casp3 | 0.547 | cytoplasm | P40223 | Csf3r | 0.605 | plasma membrane |
| P05366 | Saa1 | 0.553 | extracellular | Q9QZZ6 | Dpt | 0.61 | extracellular |

| Q91WP6 | Serpina3n | 0.561 | endoplasmic reticulum | Q61333 | Tnfaip2 | 0.617 | extracellular |
| --- | --- | --- | --- | --- | --- | --- | --- |
| P07759 | Serpina3k | 0.578 | endoplasmic reticulum | Q923T9 | Camk2g | 0.625 | cytoplasm |
| A6X935 | Itih4 | 0.581 | extracellular | B5X0G2 | Mup17 | 0.626 | extracellular |
| P05367 | Saa2 | 0.584 | extracellular | Q8VDK1 | Nit1 | 0.629 | mitochondria |
| Q8VE91 | Retreg1 | 0.595 | plasma membrane | P11798 | Camk2a | 0.633 | cytoplasm |
| Q3UNX5 | Acsm3 | 0.618 | mitochondria | Q9DBF1 | Aldh7a1 | 0.644 | plasma membrane |
| Q06890 | Clu | 0.624 | extracellular | A6X935 | Itih4 | 0.645 | extracellular |
| Q8BND5 | Qsox1 | 0.624 | Golgi apparatus | Q02819 | Nucb1 | 0.647 | extracellular |
| Q64441 | Cyp24a1 | 0.647 | mitochondria | Q64442 | Sord | 0.649 | cytoplasm |
| Q78JN3 | Eci3 | 0.657 | cytoplasm | Q7TNG8 | Ldhd | 0.66 | mitochondria |
| Q99MS3 | Mpv17l | 0.657 | mitochondria | D3YU81 | Rfx8 | 0.661 | nucleus |
