# Supplementary material for: Exogenous Biological Renal Support Improves Kidney Function in Mice With Rhabdomyolysis-Induced Acute Kidney Injury
Source: Front Med (Lausanne). 2021 May 28;8:655787. doi: 10.3389/fmed.2021.655787 (PMC8193099; doi:10.3389/fmed.2021.655787)
Supplement: Supplementary file 9 [file Data_Sheet_2.docx]

**Proteomics sample preparation**

Peripheral blood was collected and centrifuged and stored at -80 °C, followed by protein extraction. Firstly, the cellular debris of serum sample was removed by centrifugation at 12,000 g at 4 °C for 10 min. Then, the supernatant was transferred to a new centrifuge tube. The top 12 high abundance proteins were removed by Pierce™ Top 12 Abundant Protein Depletion Spin Columns Kit (Thermo Fisher). Finally, the protein concentration was determined with BCA kit according to the manufacturer’s instructions.

The frozen kidney tissue was grinded by liquid nitrogen into cell powder and then transferred to a 5-mL centrifuge tube. After that, four volumes of lysis buffer (8 M urea, 1% Protease Inhibitor Cocktail) was added to the cell powder, followed by sonication three times on ice using a high intensity ultrasonic processor (Scientz). (Note: For PTM experiments, inhibitors were also added to the lysis buffer, e.g. 3 μM TSA and 50 mM NAM for acetylation.) The remaining debris was removed by centrifugation at 12,000 g at 4 °C for 10 min. Finally, the supernatant was collected and the protein concentration was determined with BCA kit according to the manufacturer’s instructions.

**Trypsin Digestion**

For digestion, the protein solution was reduced with 5 mM dithiothreitol for 30 min at 56 °C and alkylated with 11 mM iodoacetamide for 15 min at room temperature in darkness. The protein sample was then diluted by adding 100 mM TEAB to urea concentration less than 2M. Finally, trypsin was added at 1:50 trypsin-to-protein mass ratio for the first digestion overnight and 1:100 trypsin-to-protein mass ratio for a second 4 h-digestion.

**TMT/iTRAQ Labeling**

After trypsin digestion, peptide was desalted by Strata X C18 SPE column (Phenomenex) and vacuum-dried. Peptide was reconstituted in 0.5 M TEAB and processed according to the manufacturer’s protocol for TMT kit/iTRAQ kit. Briefly, one unit of TMT/iTRAQ reagent were thawed and reconstituted in acetonitrile. The peptide mixtures were then incubated for 2 h at room temperature and pooled, desalted and dried by vacuum centrifugation.

**HPLC Fractionation**

The tryptic peptides were fractionated into fractions by high pH reverse-phase HPLC using Thermo Betasil C18 column (5 μm particles, 10 mm ID, 250 mm length). Briefly, peptides were first separated with a gradient of 8% to 32% acetonitrile (pH 9.0) over 60 min into 60 fractions. Then, the peptides were combined into 6 fractions and dried by vacuum centrifuging.

**LC-MS/MS Analysis**

The tryptic peptides were dissolved in 0.1% formic acid (solvent A), directly loaded onto a home-made reversed-phase analytical column (15-cm length, 75 μm i.d.). The gradient was comprised of an increase from 6% to 23% solvent B (0.1% formic acid in 98% acetonitrile) over 26 min, 23% to 35% in 8 min and climbing to 80% in 3 min then holding at 80% for the last 3 min, all at a constant flow rate of 400 nL/min on an EASY-nLC 1000 UPLC system.
The peptides were subjected to NSI source followed by tandem mass spectrometry (MS/MS) in Q ExactiveTM Plus (Thermo) coupled online to the UPLC. The electrospray voltage applied was 2.0 kV. The m/z scan range was 350 to 1800 for full scan, and intact peptides were detected in the Orbitrap at a resolution of 70,000. Peptides were then selected for MS/MS using NCE setting as 28 and the fragments were detected in the Orbitrap at a resolution of 17,500. A data-dependent procedure that alternated between one MS scan followed by 20 MS/MS scans with 15.0s dynamic exclusion. Automatic gain control (AGC) was set at 5E4. Fixed first mass was set as 100 m/z.

**Database Search**

The resulting MS/MS data were processed using Maxquant search engine (v.1.5.2.8). Tandem mass spectra were searched against human uniprot database concatenated with reverse decoy database. Trypsin/P was specified as cleavage enzyme allowing up to 4 missing cleavages. The mass tolerance for precursor ions was set as 20 ppm in First search and 5 ppm in Main search, and the mass tolerance for fragment ions was set as 0.02 Da. Carbamidomethyl on Cys was specified as fixed modification and acetylation modification and oxidation on Met were specified as variable modifications. FDR was adjusted to < 1% and minimum score for modified peptides was set > 40.

**Bioinformatics Methods**

**Annotation Methods**

**GO Annotation**
The Gene Ontology, or GO, is a major bioinformatics initiative to unify the representation of gene and gene product attributes across all species. More specifically, the project aims to:
1. Maintain and develop its controlled vocabulary of gene and gene product attributes;
2. Annotate genes and gene products, and assimilate and disseminate annotation data;
3. Provide tools for easy access to all aspects of the data provided by the project.
The ontology covers three domains:
1. Cellular component: A cellular component is just that, a component of a cell, but with the proviso that it is part of some larger object; this may be an anatomical structure (e.g. rough endoplasmic reticulum or nucleus) or a gene product group (e.g. ribosome, proteasome or a protein dimer).
2. Molecular function: Molecular function describes activities, such as catalytic or binding activities, that occur at the molecular level. GO molecular function terms represent activities rather than the entities (molecules or complexes) that perform the actions, and do not specify where or when, or in what context, the action takes place.
3. Biological process: A biological process is series of events accomplished by one or more ordered assemblies of molecular functions. It can be difficult to distinguish between a biological process and a molecular function, but the general rule is that a process must have more than one distinct steps.
Gene Ontology (GO) annotation proteome was derived from the UniProt-GOA database ( <http://www.ebi.ac.uk/GOA/>). Firstly, Converting identified protein ID to UniProt ID and then mapping to GO IDs by protein ID. If some identified proteins were not annotated by UniProt-GOA database, the InterProScan soft would be used to annotated protein’s GO functional based on protein sequence alignment method. Then proteins were classified by Gene Ontology annotation based on three categories: biological process, cellular component and molecular function.
**Domain Annotation**
A protein domain is a conserved part of a given protein sequence and structure that can evolve, function and exist independently of the rest of the protein chain. Each domain forms a compact three-dimensional structure and often can be independently stable and folded. Many proteins consist of several structural domains. One domain may appear in a variety of differentially expressed proteins. Molecular evolution uses domains as building blocks and these may be recombined in different arrangements to create proteins with different functions. Domains vary in length from between about 25 amino acids up to 500 amino acids in length. The shortest domains such as zinc fingers are stabilized by metal ions or disulfide bridges. Domains often form functional units, such as the calcium-binding EF hand domain of calmodulin. Because they are independently stable, domains can be “swapped” by genetic engineering between one protein and another to make chimeric proteins.
Identified proteins domain functional description were annotated by InterProScan (a sequence analysis application) based on protein sequence alignment method, and the InterPro domain database was used. InterPro (<http://www.ebi.ac.uk/interpro/>) is a database that integrates diverse information about protein families, domains and functional sites, and makes it freely available to the public via Web-based interfaces and services. Central to the database are diagnostic models, known as signatures, against which protein sequences can be searched to determine their potential function. InterPro has utility in the large-scale analysis of whole genomes and meta-genomes, as well as in characterizing individual protein sequences.
**KEGG Pathway Annotation**
KEGG connects known information on molecular interaction networks, such as pathways and complexes (the “Pathway” database), information about genes and proteins generated by genome projects (including the gene database) and information about biochemical compounds and reactions (including compound and reaction databases). These databases are different networks, known as the “protein network”, and the “chemical universe” respectively. There are efforts in progress to add to the knowledge of KEGG, including information regarding ortholog clusters in the KEGG Orthology database. KEGG Pathways mainly including: Metabolism, Genetic Information Processing, Environmental Information Processing, Cellular Processes, Rat Diseases, Drug development. Kyoto Encyclopedia of Genes and Genomes (KEGG) database was used to annotate protein pathway. Firstly, using KEGG online service tools KAAS to annotated protein’s KEGG database description. Then mapping the annotation result on the KEGG pathway database using KEGG online service tools KEGG mapper.
**Subcellular Localization**
The cells of eukaryotic organisms are elaborately subdivided into functionally distinct membrane bound compartments. Some major constituents of eukaryotic cells are: extracellular space, cytoplasm, nucleus, mitochondria, Golgi apparatus, endoplasmic reticulum (ER), peroxisome, vacuoles, cytoskeleton, nucleoplasm, nucleolus, nuclear matrix and ribosomes.
Bacteria also have subcellular localizations that can be separated when the cell is fractionated. The most common localizations referred to include the cytoplasm, the cytoplasmic membrane (also referred to as the inner membrane in Gram-negative bacteria), the cell wall (which is usually thicker in Gram-positive bacteria) and the extracellular environment. Most Gram-negative bacteria also contain an outer membrane and periplasmic space. Unlike eukaryotes, most bacteria contain no membrane-bound organelles, however there are some exceptions.
There, we used wolfpsort a subcellular localization predication soft to predict subcellular localization. Wolfpsort is an updated version of PSORT/PSORT II for the prediction of eukaryotic sequences. Special for protokaryon species, Subcellular localization prediction soft CELLO was used.

**Functional Enrichment**

**Enrichment of Gene Ontology analysis**
Proteins were classified by GO annotation into three categories: biological process, cellular compartment and molecular function. For each category, a two-tailed Fisher’s exact test was employed to test the enrichment of the differentially expressed protein against all identified proteins. The GO with a corrected p-value < 0.05 is considered significant.
**Enrichment of pathway analysis**
Encyclopedia of Genes and Genomes (KEGG) database was used to identify enriched pathways by a two-tailed Fisher’s exact test to test the enrichment of the differentially expressed protein against all identified proteins. The pathway with a corrected p-value < 0.05 was considered significant. These pathways were classified into hierarchical categories according to the KEGG website.
**Enrichment of protein domain analysis**
For each category proteins, InterPro (a resource that provides functional analysis of protein sequences by classifying them into families and predicting the presence of domains and important sites) database was researched and a two-tailed Fisher’s exact test was employed to test the enrichment of the differentially expressed protein against all identified proteins. Protein domains with a corrected p-value < 0.05 were considered significant.

**Enrichment-based Clustering**

For further hierarchical clustering based on differentially expressed protein functional classification (such as: GO, Domain, Pathway, Complex). We first collated all the categories obtained after enrichment along with their P values, and then filtered for those categories which were at least enriched in one of the clusters with P value <0.05. This filtered P value matrix was transformed by the function x = −log10 (P value). Finally these x values were z-transformed for each functional category. These z scores were then clustered by one-way hierarchical clustering (Euclidean distance, average linkage clustering) in Genesis. Cluster membership were visualized by a heat map using the “heatmap.2” function from the “gplots” R-package.

**Protein-protein Interaction Network**

All differentially expressed protein database accession or sequence were searched against the STRING database version 10.1 for protein-protein interactions. Only interactions between the proteins belonging to the searched data set were selected, thereby excluding external candidates. STRING defines a metric called “confidence score” to define interaction confidence; we fetched all interactions that had a confidence score ≥ 0.7 (high confidence). Interaction network form STRING was visualized in R package “networkD3”.

The Venn's diagrams was performed by the online sofeware (Oliveros, J.C. (2007-2015) Venny. An interactive tool for comparing lists with Venn's diagrams. https://bioinfogp.cnb.csic.es/tools/venny/index.html).
